# Pan-histone deacetylase inhibitor vorinostat suppresses osteoclastic bone resorption through modulation of RANKL-evoked signaling and ameliorates ovariectomy-induced bone loss

**DOI:** 10.1186/s12964-024-01525-w

**Published:** 2024-03-04

**Authors:** Xiaole Peng, Tianhao Wang, Qing Wang, Yuhu Zhao, Hao Xu, Huilin Yang, Ye Gu, Yunxia Tao, Bangsheng Yan, Yaozeng Xu, Dechun Geng

**Affiliations:** 1 Department of Orthopedics, The First Affiliated Hospital of Soochow University, 188 Shizi Street, Suzhou, 215006 Jiangsu China; 2 Department of Orthopedics, Wuxi 9th People’s Hospital Affiliated to Soochow University, Wuxi, 214000 Jiangsu China; 3 Department of Orthopedics, Changshu First People’s Hospital Affiliated to Soochow University, Changshu, 215500 Jiangsu China; 4 Department of Orthopedics, Huishan Second People’s Hospital, Wuxi, 214174 China

**Keywords:** Vorinostat, Osteoporosis, Osteoclastogenesis, RANKL

## Abstract

**Background:**

Estrogen deficiency-mediated hyperactive osteoclast represents the leading role during the onset of postmenopausal osteoporosis. The activation of a series of signaling cascades triggered by RANKL-RANK interaction is crucial mechanism underlying osteoclastogenesis. Vorinostat (SAHA) is a broad-spectrum pan-histone deacetylase inhibitor (HDACi) and its effect on osteoporosis remains elusive.

**Methods:**

The effects of SAHA on osteoclast maturation and bone resorptive activity were evaluated using in vitro osteoclastogenesis assay. To investigate the effect of SAHA on the osteoclast gene networks during osteoclast differentiation, we performed high-throughput transcriptome sequencing. Molecular docking and the assessment of RANKL-induced signaling cascades were conducted to confirm the underlying regulatory mechanism of SAHA on the action of RANKL-activated osteoclasts. Finally, we took advantage of a mouse model of estrogen-deficient osteoporosis to explore the clinical potential of SAHA.

**Results:**

We showed here that SAHA suppressed RANKL-induced osteoclast differentiation concentration-dependently and disrupted osteoclastic bone resorption in vitro. Mechanistically, SAHA specifically bound to the predicted binding site of RANKL and blunt the interaction between RANKL and RANK. Then, by interfering with downstream NF-κB and MAPK signaling pathway activation, SAHA negatively regulated the activity of NFATc1, thus resulting in a significant reduction of osteoclast-specific gene transcripts and functional osteoclast-related protein expression. Moreover, we found a significant anti-osteoporotic role of SAHA in ovariectomized mice, which was probably realized through the inhibition of osteoclast formation and hyperactivation.

**Conclusion:**

These data reveal a high affinity between SAHA and RANKL, which results in blockade of RANKL-RANK interaction and thereby interferes with RANKL-induced signaling cascades and osteoclastic bone resorption, supporting a novel strategy for SAHA application as a promising therapeutic agent for osteoporosis.

**Supplementary Information:**

The online version contains supplementary material available at 10.1186/s12964-024-01525-w.

## Background

Osteoporosis, particularly postmenopausal osteoporosis (PMOP), is a common age-related skeletal disorder characterized by a systematic deterioration of bone mass, bone strength, and bone microarchitecture [1]. In fact, osteoporosis is a near-universal process in individuals past middle age. With an aging population, osteoporosis and the possible accompanying fragility fractures have emerged as a major public health problem, imposing an enormous medical and socioeconomic impact [2]. From 2010 to 2050, it is predicted that osteoporotic fractures in China will increase by 257% (2.33–5.99 million), and the socioeconomic burden will grow from $9.45 billion to $25.43 billion annually [3]. The prevalence of osteoporosis in women over the age of 50 years is about three times that in men [4]. The lifetime risk of any osteoporotic fracture in a 50-year-old woman is as high as 50% [5, 6]. One of the major predisposing factors for the pathogenesis of osteoporosis in women is menopause [7]. With constant bone remodeling, the coupling of osteoblastic bone formation and osteoclastic bone resorption determines bone mass and the balance of bone metabolism. Estrogen deficiency disrupts this homeostasis and consequently provokes accelerated postmenopausal bone loss [8, 9]. Conventional estrogen replacement is no longer recommended for the prevention and treatment of PMOP due to its own increased risks such as breast cancer and cardiovascular and cerebrovascular events [10]. The past 30 years have witnessed tremendous progress in the development and clinical practice of anti-osteoporotic drugs. Nevertheless, in view of the inconclusive long-term efficacy and significant, albeit rare, complications associated with the use of currently approved osteoporosis therapies [11, 12], compelling reasons exist for continued new drug discovery to treat the disease and prevent fractures.

Osteoclasts are giant multinucleated cells derived from the monocyte/macrophage lineage of hematopoietic origin. They are the only bone cells responsible for bone resorption and characteristically secrete specific protein markers like tartrate-resistant acid phosphatase (TRAcP) [13]. The presence of receptor activator of nuclear factor κB (NF-κB) ligand (RANKL) is fundamental with respect to osteoclast formation and survival [14]. Through interacting with its receptor RANK, RANKL exposure initiates the recruitment of TNF receptor associated factor 6 (TRAF6) and regulates a series of downstream targets including NF-κB and mitogen-activated protein kinases (MAPK) signaling pathways, leading to the auto-amplification of nuclear factor of activated T cells c1 (NFATc1) [14, 15]. Upregulated NFATc1 nuclear expression promotes osteoclast-specific gene transcription, which consequently results in osteoclast maturation [16, 17]. Enhanced osteoclastogenesis due to estrogen withdrawal at menopause, manifested by excessive bone resorption and relatively defective bone formation, is the most fundamental characteristic of PMOP [8]. Accordingly, the suppression of osteoclast hyperactivation through blockade of RANKL-induced signaling represents an attractive and promising candidate therapeutic protocol for estrogen-deficient osteoporosis.

Histone deacetylase (HDAC) inhibitors (HDACi) exhibit excellent in vivo anti-cancer efficacy due to their suppressive ability on cancer cell migration, invasion, metastasis, and angiogenesis [18]. In addition to their prevalence in cancer therapeutics, HDACi have recently aroused widespread interest within other disorder-related fields [19] and emerged as prospective agents to inhibit osteoclastogenesis and treat osteolytic diseases like periprosthetic osteolysis [20], periodontitis [21] and rheumatoid arthritis (RA) [22]. As the first FDA-approved HDACi for treating cutaneous T-cell lymphoma (CTCL), vorinostat (suberoylanilide hydroxamic acid; SAHA) has proven its superior efficacy in a variety of tumors and holds great practical value in clinical oncology therapeutics [23]. Moreover, SAHA has been reported to exert immunosuppressive function and has promising applications in the treatment of arthritis disorders [24, 25]. As a broad-spectrum pan-HDACi, SAHA suppresses the deacetylase activity of class I and II HDACs at low nanomolar concentrations, and thus, influences gene transcription by eliciting dynamic shifts in the acetylation and deacetylation status of key proteins [26]. Accumulating evidence reveals a strong association of class I and II HDACs with proper skeletal development [27]. Although SAHA has been demonstrated to inhibit osteoclastogenesis in vitro [28], its underlying molecular mechanism requires further elucidation. Furthermore, the therapeutic effect of SAHA on osteoporotic bone loss mediated by estrogen deficiency remains unknown.

Given the crucial roles of RANKL-induced signaling in osteoclast differentiation and maturation and the potential therapeutic applications of HDACi in skeletal diseases, we hypothesized that SAHA might ameliorate osteoporotic bone loss by interfering with osteoclast overactivation. In this study, we demonstrated that SAHA impaired the binding of RANKL to RANK and blocked the downstream NF-κB and MAPK pathways, thus negatively regulating RANKL-induced osteoclastogenesis. In vivo, our findings revealed that SAHA administration protected mice from ovariectomy-induced bone loss.

## Methods

### Reagents and media

MedChemExpress (New Jersey, USA) provided the high purity (≥99.0%) SAHA used in this study. Dimethyl sulfoxide (DMSO) (Sigma–Aldrich, Sydney, NSW, Australia) was employed to prepare SAHA to a concentration of 10 mM for storage at − 20 °C before being diluted to working concentrations with culture medium. Dulbecco’s modified Eagle’s medium (DMEM/high glucose) along with fetal bovine serum (FBS) were acquired from HyClone (Logan, UT, USA), whereas recombinant murine RANKL was supplied by R&D system (Minneapolis, MN, USA). FITC-phalloidin was procured from Thermo Fisher Scientific (Scoresby, VIC, Australia) and DAPI staining solution was bought from Beyotime (Shanghai, China). Primary antibodies against β-Actin (AC006), NFATc1 (A1539), CTSK (A5871), MMP9 (A11147), phospho-P38 (AP0057), phospho-ERK (AP0485), ERK (A4782), and phosphor-P65 (AP0475) were obtained from ABclonal (Wuhan, China). Primary antibody against IκB-α (AF5002) was obtained from Affinity Biosciences (Jiangsu, China). Primary antibodies for P38 (ab170099), phospho-JNK (ab76572), JNK (ab208035), and TRAF6 (ab40675) were purchased from Abcam (Cambridge, UK). The corresponding secondary antibodies were purchased from Beyotime (Shanghai, China). Yangming Biotechnology (Hangzhou, China) supplied bovine bone slices.

### Cell culture and osteoclast differentiation

Bone marrow-derived macrophages (BMDM) were prepared as reported in our previous study [29]. Briefly, BMDM were harvested from the femur bone marrow of C57BL/6 (6–8 weeks of age) mice and cultured in α-MEM with 10% FBS, 1% penicillin-streptomycin, and 25 ng/mL macrophage colony-stimulating factor (M-CSF) after lysing the red blood cells. The cells were incubated for 4 days with the medium changed every other day. Then, BMDM adhering to the bottom of the culture dish were collected and cultured for an additional 6 days in the presence of M-CSF (25 ng/mL) and RANKL (50 ng/mL) to induce osteoclast differentiation. The RAW 264.7 murine macrophage cell line was offered by the Cell Bank of the Chinese Academy of Sciences (Shanghai, China) and cultivated in DMEM supplemented with 10% FBS under 5% CO_2_ at 37 °C. For in vitro osteoclast differentiation experiments, RAW 264.7 cells were seeded into 48-well plates at a density of 1 × 10^4^ cells per well and cultured overnight to allow adherence. Cells were stimulated the next day with RANKL (50 ng/ml) and given escalating doses of SAHA (0, 0.1, 0.25, 0.5, 1 μM). The culture medium was renewed every two days until multinucleated cell formation. To investigate the influence of SAHA on osteoclasts at different stages of differentiation, SAHA (1 μM) was used for intervening RAW 264.7 cells at distinct time courses (early, middle, and late stages) during 7-day cell culture.

The cells were stained with a TRAcP staining Kit (BZ Biotechnology, Jiangsu, China) based on the manufacturer’s protocol after washing with phosphate-buffered saline (PBS) and fixing with 4% paraformaldehyde (PFA) (Biosharp, Anhui, China). The fused cells exhibiting three or more nuclei were counted as mature osteoclasts. Images were captured by an inverted microscope (Zeiss, Dresden, Germany) and quantification was conducted utilizing ImageJ software (National Institutes of Health, MD, USA).

### Cytotoxicity assay

The cytotoxicity of SAHA on RAW 264.7 cells or BMDM cells at a range of doses was evaluated using a Cell Counting Kit-8 (CCK-8) (Beyotime) in accordance with the manufacturer’s instructions. Cells were nurtured at a density of 6 × 10^3^ RAW 264.7 cells or 1.2 × 10^4^ BMDM cells per well in 96-well plates with the appropriate culture medium. After 12 h, distinct concentrations of SAHA were added to each well and cultivated for 72 h. Afterwards, the cells were incubated with culture medium containing 10% CCK-8 solution for an additional 2 h at 37 °C in the dark. The absorbance of each well at 450 nm was finally measured using a microplate reader.

### Immunocytochemistry

The effects of SAHA on F-actin ring formation and NFATc1 activity were assessed through using the method of immunofluorescence labeling on RANKL-stimulated osteoclasts under SAHA (0, 0.5, 1 μM) intervention for 5 days. Cells were fixed with chilled 4% PFA (Biosharp) with subsequent permeabilization with 0.1% Triton X-100 (Beyotime) and blocking with QuickBlock™ blocking buffer (Beyotime). After co-incubation with anti-NFATc1 primary antibody (1:250) at 4 °C for 16 h, cells were rinsed with PBS and incubated with the corresponding fluorescent secondary antibody (conjugated to Alexa Fluor 647, red, Abcam). FITC-phalloidin was utilized to stain F-actin belt through incubation for 1 h in the dark, while cell nuclei were stained with DAPI for 10 min after washing with PBS. Visualization was achieved with a fluorescence microscope (Zeiss), and the mean fluorescence intensity was measured using ImageJ.

### Bone pit resorption assay

RAW 264.7 cells were seeded onto bovine bone slices in 24-well plates at a frequency of 2 × 10^5^ cells/well and stimulated with 50 ng/ml RANKL to obtain mature osteoclasts. Cells were intervened with SAHA (0, 0.5, 1 μM) for 3 days after tiny osteoclasts were shaped. The bone resorption pits on each group of slices were photographed by a scanning electron microscope (SEM) (GeminiSEM300, ZEISS, Germany) and quantitated using ImageJ software.

### High-throughput transcriptome sequencing (RNA-seq)

RNA-seq and data analysis were performed by Azenta Co., Ltd. (Suzhou, China) and were used to identify mRNA transcripts with differential expression between RANKL and SAHA (1 μM) co-stimulated RAW 264.7 cells for 3 days and positive control for RANKL exposure only. RNA preparation and sequencing library preparation for RNA-seq were constructed according to manufacturer’s instructions. The DESeq2 Bioconductor package (version 1.26.0) was utilized for differential expression analysis [30]. Statistically significant differential transcript expression was defined according to the thresholds |Log2 Fc| > 1 and the adjusted *P* value < 0.05. Insights into the changes in phenotype were visualized by Kyoto Encyclopedia of Genes and Genomes (KEGG) [31] and Gene Ontology (GO) enrichment analysis using GOSeq (Version 1.34.1) [32]. Each treatment contained three biological replicates.

### Quantitative real-time polymerase chain reaction (qRT–PCR)

Total cellular RNA was isolated from samples using TRIzol reagent (Beyotime). Then, with 1 μg of total RNA as the initial template, reverse transcription was implemented to synthesize complementary DNA (cDNA). qRT–PCR testing was carried out in a CFX96™ thermal cycler (Bio- Rad Laboratories) by mixing cDNA proportionally with SYBR Green PCR Master Mix (Yeasen, Shanghai, China) and corresponding primers. The specific primers’ information was described in Additional file 1: Table S1. Relative mRNA expression of each group was normalized to the average expression of *Actb* and calculated on the basis of the 2^−ΔΔCq^ method.

### Western blot analysis

At the scheduled time points after incubation, cellular proteins in samples were extracted by lysing with radioimmunoprecipitation assay (RIPA; Beyotime) buffer. Proteins (20 μg) were resolved by SDS–PAGE (Beyotime) and transferred to nitrocellulose membranes (Beyotime). After closure with 5% skim milk or 5% BSA for at least 1 h, membranes were incubated separately with primary antibodies against β-Actin (1:1000), NFATc1 (1:1000), MMP9 (1:1000), CTSK (1:1000), IκB-α (1:1000), phospho-P38 (1:1000), P38 (1:2500), phospho-JNK (1:5000), JNK (1:2000), phospho-ERK (1:1000), ERK (1:1000) and TRAF6 (1:5000) at 4 °C overnight. The corresponding secondary antibody dilutions (1:1000) were then added for 1 h at room temperature after washing membranes adequately with Tris-buffered saline Tween (TBST) for an appropriate time and frequency. Finally, to show the protein bands, membranes were treated with enhanced chemiluminescence (ECL; Yeasen) reagents and imaged using Image Lab 3.0. Protein quantification (relative grey level of the bands) was measured using ImageJ software.

### Molecular docking

The SDF format of SAHA was acquired from PubChem and subsequently converted to MOL2 format via OpenBabel software (version 2.4.1). To determine the affinity and binding mode of the SAHA-RANKL complex, molecular docking was carried out using Autodock4 (version 4.2.6). The crystal structure of RANK bound to RANKL (ID: 4GIQ) was retrieved from Protein Data Bank (PDB). The structures of both protein and macromolecule were converted to PDBQT format prior to computational docking. The optimized structure of RANKL was conducted through removing the water molecules, adding hydrogens, and minimizing energy. Then, a grid box containing all possible binding sites for the docking stage of compound was selected. We eventually acquired the docking pose of SAHA on RANKL with the highest-scoring conformation and visualized the results using PyMOL (version 2.2.0) to display the potential interactive residues. A 2D interaction of RANKL protein and SAHA ligand was generated in LigPlot+ (version 2.2) [33].

### Ovariectomy (OVX)-induced osteoporosis mouse model

A total of forty 8-week-old female C57BL/6 J mice were supplied by the Animal Experiment Center of Soochow University. Prior to operation, mice were housed in appropriate temperature and light conditions for one week to acclimate to the environment. All mice were randomly assigned to four groups (*n* = 10 per group): sham group, OVX group (bilateral ovariectomy), OVX + Low-SAHA (25 mg/kg) group, and OVX + High-SAHA (50 mg/kg) group. OVX mice were subjected to bilateral ovariectomy under pentobarbital anesthesia to induce osteoporosis, while normal control mice underwent sham operation in which the ovaries were only exteriorized without resection. After one-week recovery, the mice in the OVX + Low-SAHA group and OVX + High-SAHA group were administered SAHA at doses of 25 mg/kg and 50 mg/kg intraperitoneally every other day for six weeks, respectively. The mice in the sham and OVX groups were injected intraperitoneally with the same volume of vehicle (5% DMSO dissolved in solution of 40% PEG300 + 5% Tween-80 + 50% saline) as a control. After six-week injection, all mice were euthanized. Femur specimens were taken for micro-CT and bone histomorphometry analysis, blood samples were harvested for enzyme-linked immunosorbent assay (ELISA) testing, and liver, kidney, heart and lung tissues were taken for drug toxicity assessment. The dose and frequency of SAHA administration were determined based on previous literature [34–37].

### ELISA testing

To explore the interference of SAHA with RANK-RANKL interaction, preosteoclasts RAW 264.7 cells were seeded on 6-well plates at a density of 2 × 10^5^ cells per well and cultured overnight at 37 °C. Cells were then incubated with RANKL (50 ng/ml) and varying doses of SAHA (0, 0.5, and 1 μM) for 72 h. Cell culture medium were collected and centrifuged at 2500 rpm for 20 min at 4 °C to remove cells and impurities. The RANKL-scavenging efficiency mediated by cell surface receptor RANK was evaluated through measuring the RANKL concentration in the supernatant using an ELISA kit (LunChangShuoBiotech, Xiamen, China).

Mouse serum samples were prepared from blood samples after standing for 1 h at room temperature and centrifugation at 3000 rpm for 15 min. The serum levels of bone resorption biomarkers, TRAcP-5b and type I collagen cross-linked C-terminal telopeptide (β-CTx), were estimated using the corresponding ELISA kit (Elabscience, Wuhan, China).

### Micro-CT scanning

The micro-architecture of mouse femurs was scanned with a SkyScan 1176 micro-CT instrument (Kontich, Belgium). Then, the 2D and 3D original images were reconstructed by DataViewer and NRecon software (Kontich, Belgium), respectively. The region of interest (ROI) of trabecular bone was set 0.6 mm above the growth plate of the distal femur and 1.5 mm in height, while the ROI of cortical bone was generated in the mid shaft (3 mm from the base of the growth plate) with a height of 1 mm. Structural parameters within the ROI were analyzed using CTAn software (Kontich, Belgium). The trabecular bone relevant parameters including bone mineral density (BMD), the trabecular bone volume to total bone volume ratio (BV/TV), trabecular number (Tb,N), and trabecular separation (Tb.Sp) as well as the cortical bone relevant parameters including BMD, cortical thickness (Ct.Th), and cortical bone area (Ct.Ar) were presented in this study.

### Femur histomorphometry analysis

Murine femurs were fixed in 10% neutral buffered formalin for at least 48 h and decalcified in 10% EDTA (Sigma–Aldrich) for 1 month until soft, following by embedded in paraffin and sliced into 6-μm-thick sections (Leica 2135, Germany). H&E staining was conducted to visualize morphological variations and evaluate bone volume. TRAcP staining was applied to observe osteoclast distribution and evaluate osteoclast activity. The images of these femoral slices were captured with an Axiovert 40C optical microscope (Zeiss, Germany). The visceral organ tissues of mice from each group were also paraffin-embedded, sectioned separately, and subjected to H&E staining, in order to assess the in vivo toxicity of SAHA.

### Immunohistochemistry (IHC)

Tissue sections were deparaffinized in xylene and rehydrated with gradient alcohol, followed by antigen repair using citrate buffer. The serum-blocked sections were then incubated with the required primary antibody dilutions, including TRAF6 (1:50), phospho-P65 (1:200), and phosphor-ERK (1:200), overnight at 4 °C. Thereafter, the sections were sequentially incubated with biotin-labeled secondary antibodies of goat anti-rabbit IgG and streptavidin-conjugated peroxidase for 30 min at 37 °C. The chromogenic reaction was induced using DAB (Cell Signaling Technology, Danvers, MA, USA) under a microscope. ImageJ software was utilized to assess positively stained cells in random regions.

### Statistical analysis

All quantitative data were displayed as mean ± standard deviation (SD) and were analyzed with GraphPad Prism software version 8.0 (GraphPad Software, San Diego, CA, USA). Statistical significance was determined by one-way or two-way analysis of variance (ANOVA) with Tukey’s test, with a *p* value < 0.05 regarded as significant.

## Results

### SAHA attenuates RANKL-mediated osteoclastogenesis in vitro

**Fig. 1:**
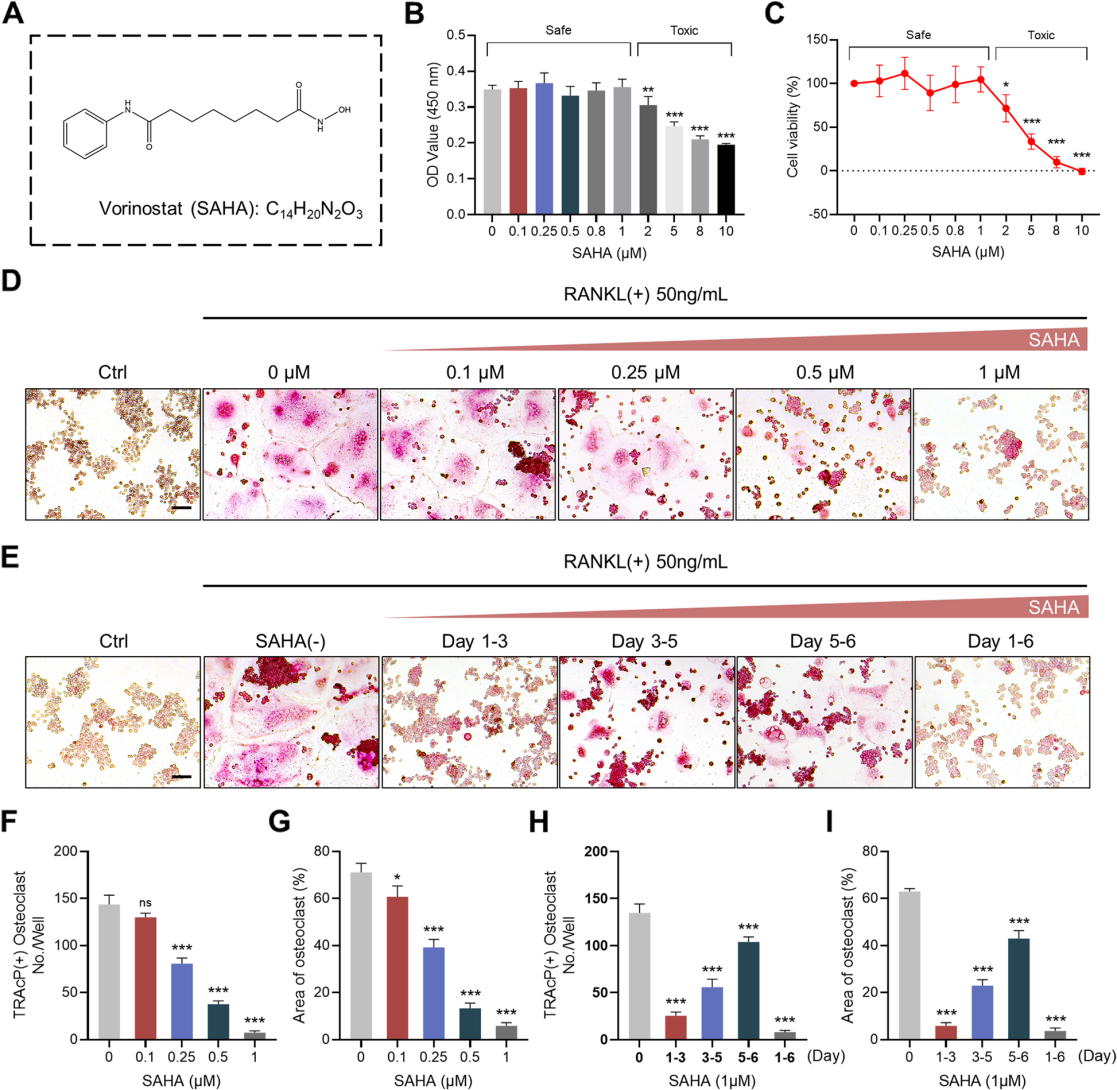
SAHA restrains RANKL-induced osteoclast differentiation. **A** The chemical structure and molecular formula of SAHA. **B-C** The effects of distinct SAHA doses on RAW 264.7 cell viability for 72 h as evaluated by CCK-8 assay. **D** Representative images of TRAcP staining visualizing the formed osteoclasts treated with SAHA at indicated doses (Scale bar = 100 μm). **E** Representative TRAcP staining images displaying osteoclasts treated with 1 μM SAHA at different stages (Scale bar = 100 μm). **F-G** Quantification of TRAcP+ multinucleate cells (nuclei > 3) per field and the average area of osteoclasts per group in Fig. 1D. **H-I** Quantitative analysis of TRAcP+ multinucleated cells with nuclei count greater than 3 and area of osteoclasts in Fig. 1D. Bar graphs are shown as mean ± SD, *n* = 3 per group. *p < 0.05, **p < 0.01, ***p < 0.001 and ns: no significance, as compared to the positive control group (treated with RANKL but without SAHA)

The chemical structure and formula of SAHA were illustrated in Fig. 1A. To clarify whether cell cytotoxicity might be implicated in the inhibition of osteoclastogenesis, a CCK-8 assay was applied to assess cell viability. As shown in Fig. 1B and C, SAHA at concentrations lower than 2 μM had no potential cytotoxicity to RAW 264.7 cells. SAHA was found to restrain cell proliferation when its concentrations were 2-10 μM. Then, to clarify the effect of SAHA on osteoclast formation in vitro, RAW 264.7 cells treated with 50 ng/ml RANKL were incubated with varying dosages of SAHA for the specific time. The formation of abundant TRAcP+ multinucleated osteoclasts was observed in the positive control group (without SAHA), whereas the cell size and the amount of TRAcP+ osteoclasts gradually diminished after intervention with increasing concentrations of SAHA (Fig. 1D, F and G). These results demonstrated that SAHA attenuated RANKL-stimulated osteoclast formation in a concentration-dependent manner at 0.1 to 1 μM. To further examine which stage of osteoclastogenesis was impacted by SAHA treatment, RANKL-induced cells were exposed to SAHA for indicated time intervals. SAHA was noted to markedly inhibit oteoclastogenesis primarily in the early stage (Day 1-3) of differentiation (Fig. 1E, H and I).

### SAHA restricts F-actin belt formation and the osteoclastic bone resorptive activity

**Fig. 2:**
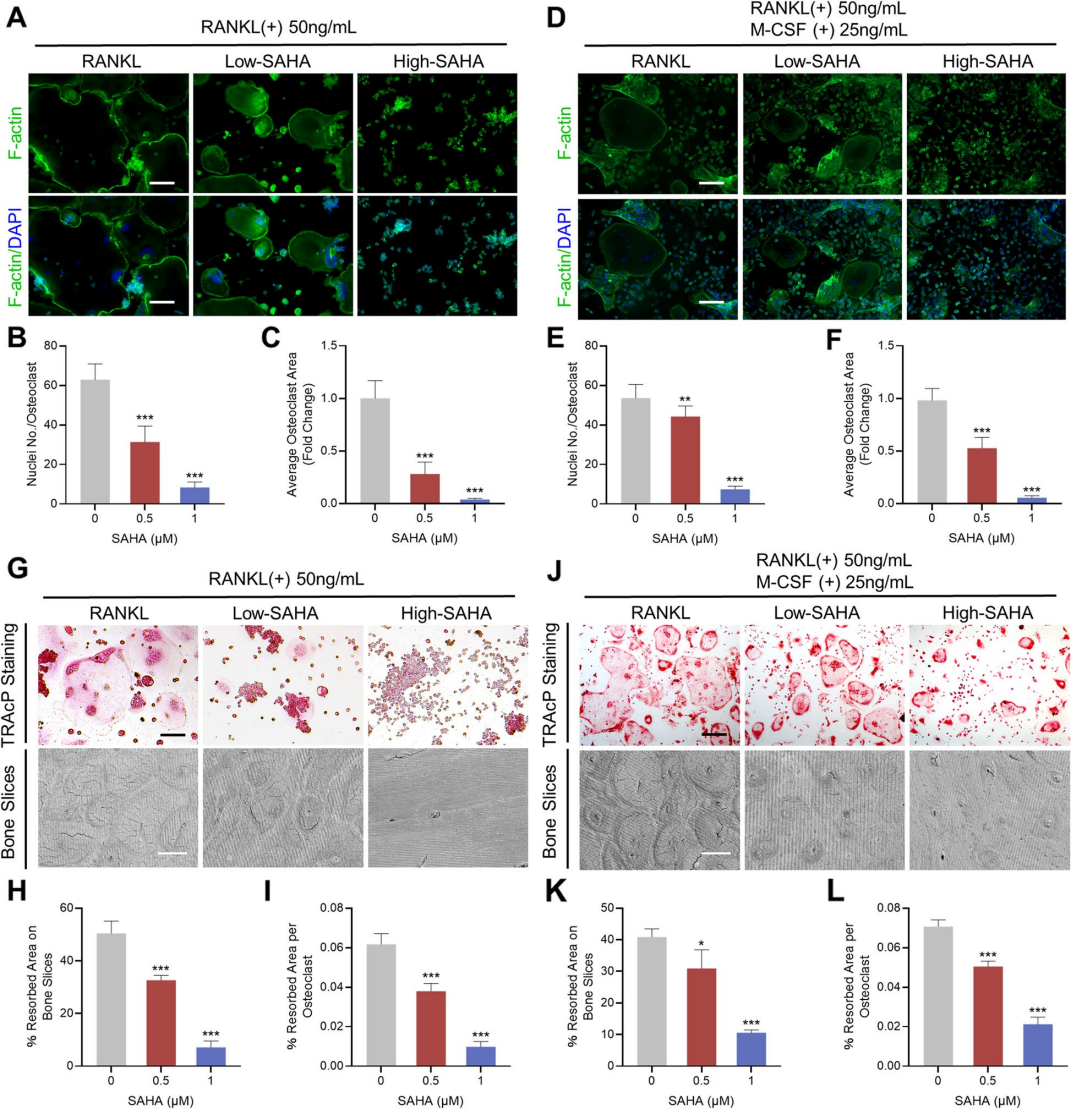
SAHA impedes F-actin belt formation and osteoclastic bone resorption. **A** Representative fluorescence micrographs for F-actin belt formation after SAHA addition in RAW 264.7 cells (Scale bar = 100 μm). F-actin (green color) and nuclei (blue color). **B-C** Quantitative statistics of the nuclei number per osteoclast and mean area of osteoclasts. **D** Representative fluorescence micrographs for F-actin belt formation after SAHA addition in BMDM (Scale bar = 100 μm). F-actin (green color) and nuclei (blue color). **E-F** Quantitative statistics of the nuclei number per osteoclast and mean area of osteoclasts. **G** Representative images of TRAcP staining for osteoclast maturation after treating SAHA in RAW 264.7 cells (Scale bar = 100 μm) and electron microscope scanning of bovine bone slices resorption pits (Scale bar = 100 μm). **H-I** Quantification of the average area of bone slice resorption and the area of resorption per osteoclast in each group. **J** Representative images of TRAcP staining for osteoclast maturation after treating SAHA in BMDM (Scale bar = 100 μm) and electron microscope scanning of bovine bone slices resorption pits (Scale bar = 100 μm). **K-L** Quantification of the average area of bone slice resorption and the area of resorption per osteoclast in each group. Bar graphs are indicated by mean ± SD, *n* = 3 per group. *p < 0.05, **p < 0.01, ***p < 0.001, in comparison to the positive control group (treated with RANKL but without SAHA)

Mature osteoclasts organize their cytoskeleton to form a filamentous actin (F-actin) belt, which is indispensable for bone resorption [38]. The F-actin belt and nucleus of osteoclasts intervened with SAHA (representative concentrations of 0.5 and 1 μM) were stained with FITC phalloidin and DAPI staining solution respectively. As shown in Fig. 2A–C, the formation of giant and well-delineated F-actin belts accompanied by numerous nuclei in the periphery of mature osteoclasts was observed in response to the stimulus of RANKL, whereas SAHA application (0.5 or 1 μM) reduced osteoclast size and nuclear number in a concentration-dependent manner. To define whether SAHA hinders osteoclast maturation in the mouse primary cell line BMDM, the inhibitory effects of different doses of SAHA on BMDM were first evaluated. Consistent with observations in RAW 264.7 cells, CCK-8 assay results indicated that SAHA have evident cytotoxicity to BMDM cells at concentrations of 2 μM and higher than 2 μM (Additional file 2: Fig. S1). Addition of 0.5 or 1 μM SAHA also restricted F-actin belt formation in BMDM without affecting cellular growth and survival and the differences were statistically significant (Fig. 2D-F).

We next utilized SEM observation to further examine the role of SAHA on the resorptive activity of mature osteoclasts in vitro. RAW 264.7 cells and BMDM were incubated with RANKL, respectively, until the formation of massive multinucleated osteoclasts, and equal numbers of cells were simultaneously seeded onto bovine bone slices. SAHA intervention induced a dose-dependent decline in osteoclast number and bone resorption area represented by resorption pits on the bone slices (Fig. 2G–L). These findings indicate that SAHA suppresses RANKL-induced osteoclast formation in vitro, resulting in a restrained resorptive function of mature osteoclasts.

### SAHA alters osteoclast gene networks during RANKL-induced osteoclastogenesis

To explore the mechanistic role of SAHA in osteoclast biological behavior, we conducted in-depth analysis of SAHA in terms of its effect on the genomic transcriptional network of osteoclasts. Whole-transcriptome RNA sequencing (RNA-seq) and bioinformatics analysis were performed on SAHA-treated cells after RANKL-induced osteoclastogenesis versus control cells with RANKL induction only.

**Fig. 3:**
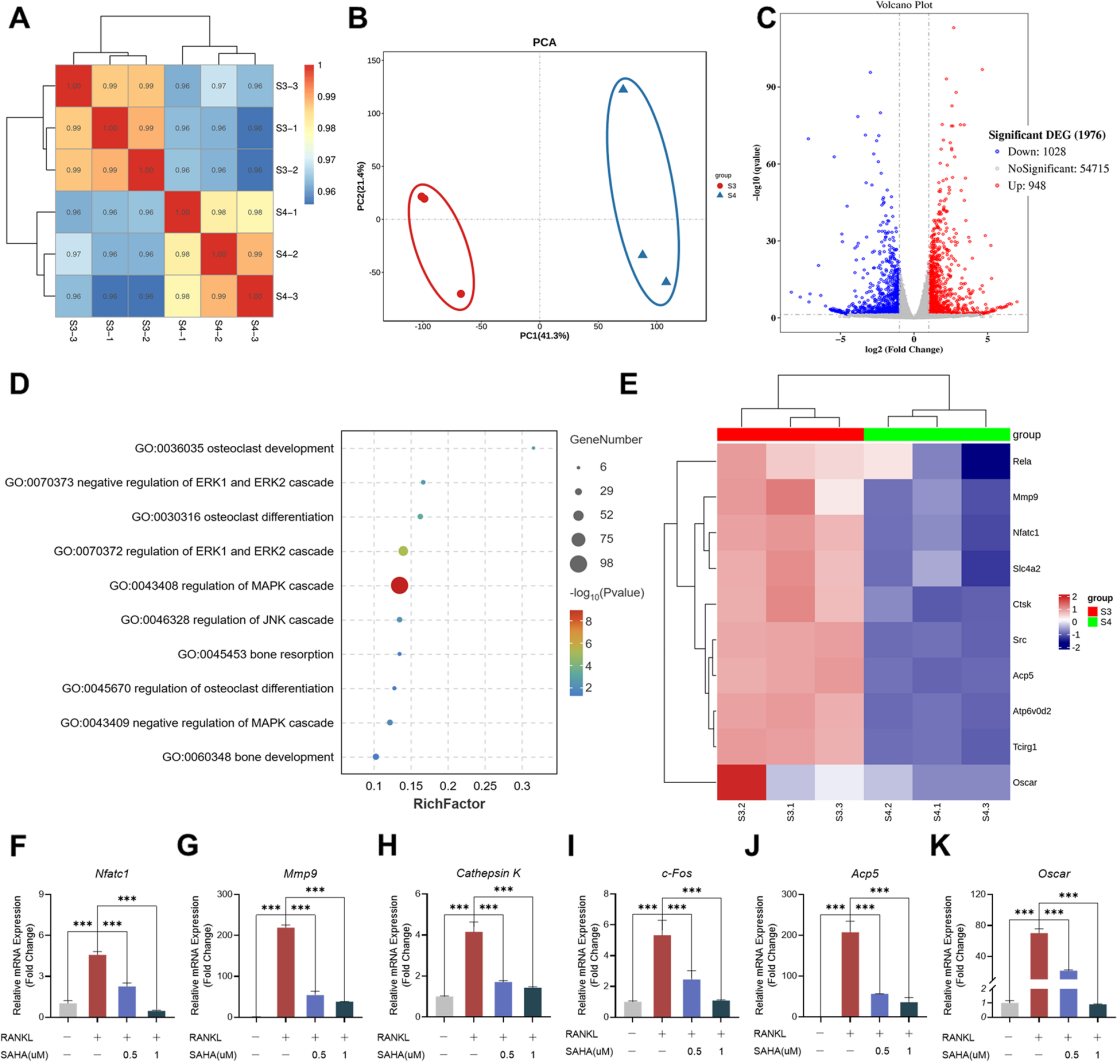
SAHA alters osteoclast gene networks and downregulates osteoclast-specific gene expression. RNA-seq was conducted with the following two groups (3 replicates per group): S3 (RANKL-induced osteoclasts) and S4 (RANKL-induced osteoclasts after SAHA treatment). **A** Pearson correlation coefficient analysis of high-throughput RNA-seq samples. **B** PCA plot of RNA-seq samples. **C** Volcano plot illustrating the differential expression of genes after treating with SAHA. Statistically significantly upregulated genes appear in red and downregulated genes appear in blue. **D** GO analysis bubble chart of osteoclastogenesis-associated gene expression profiles. **E** Gene heat map of osteoclastic differentiation-related genes. **F-K** qRT–PCR analysis of mRNA expression of *Nfatc1*, *Mmp9*, *Cathepsin K*, *c-Fos*, *Acp5* and *Oscar*

Pearson correlation coefficient analysis and principal component analysis (PCA) plot revealed high correlation and great reproducibility of the samples for sequencing (Fig. 3A and B). A total of 1976 differentially expressed genes (DEGs) between the SAHA-treated group and control group were identified, among which 948 were upregulated and 1028 were downregulated (Fig. 3C). GO analysis was performed to enrich these SAHA-regulated functional DEGs, with a focus on highly osteoclastogenesis-associated GO items according to the enrichment level. As illustrated in Fig. 3D, DEGs were remarkably enriched in osteoclastogenesis-associated biological processes, such as osteoclast development, differentiation and bone resorption as well as MAPK signal transduction, suggesting that SAHA intervention significantly affected RANKL-stimulated osteoclastogenesis. The gene expression profiles showed significant downregulation of several osteoclastic differentiation-related genes such as *Nfatc1, Acp5, Atp6v0d2, Cathepsin K (Ctsk), and Mmp9* (Fig. 3E)*.* We then performed qRT-PCR analysis for further validation. As displayed in Fig. 3F-K, Nfatc1, Mmp9, Cathepsin K, c-Fos, Acp5 and Oscar genes were all intensively expressed during RANKL-mediated osteoclastogenesis, whereas SAHA diminished their expression levels at concentrations of 0.5 μM and 1 μM, suggesting an adverse effect of SAHA on RANKL-induced expression of specific osteoclast-associated genes.

### SAHA abrogates NFATc1 signaling

**Fig. 4:**
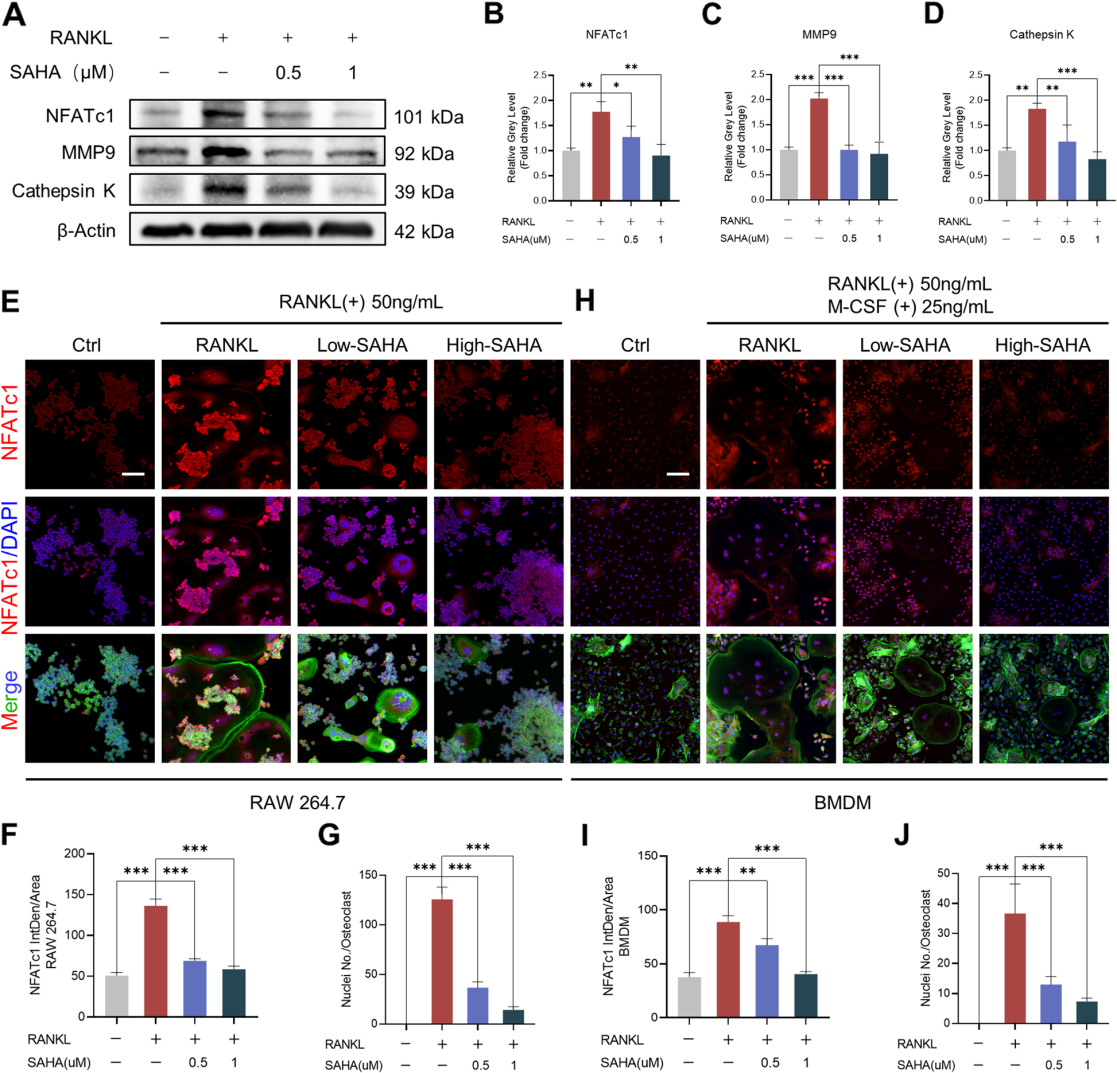
SAHA inhibits NFATc1 signaling. **A** The expression of NFATc1, MMP9 and Cathepsin K proteins. **B-D** Quantitative statistics of NFATc1, MMP9 and Cathepsin K expression normalized to β-actin expression. **E** Representative images of immunofluorescence staining indicating the effect of SAHA on RANKL-induced activity of NFATc1 in RAW 264.7 cells (Scale bar = 100 μm). NFATc1 (red color), F-actin (green color) and nuclei (blue color). **F-G** Quantification of the mean fluorescence intensity of NFATc1 and the nuclei number per osteoclast. **H** Representative images of immunofluorescence staining indicating the effect of SAHA on RANKL-induced activity of NFATc1 in BMDM (Scale bar = 100 μm). NFATc1 (red color), F-actin (green color) and nuclei (blue color). **I-J** Quantification of the mean fluorescence intensity of NFATc1 and the nuclei number per osteoclast. All data are shown as mean ± SD, *n* = 3 per group. *p < 0.05, **p < 0.01, ***p < 0.001, relative to the positive control group (treated with RANKL but without SAHA)

To further validate the effect of SAHA on osteoclast differentiation inhibition, the translational levels of key proteins implicated in osteoclastogenesis were determined. Our results of western blot indicated that the sustained expression of NFATc1, a crucial transcription factor in osteoclastogenesis, and osteo-resorptive proteins (MMP9 and Cathepsin K) after RANKL exposure were restrained following SAHA addition at doses of 0.5 μM and 1 μM (Fig. 4A-D). Moreover, we measured NFATc1 activity in RAW 264.7 cells by using immunofluorescence staining. As shown in Fig. 4E-G, the expression of NFATc1 was dramatically increased under RANKL exposure accompanied by the formation of giant multinucleated osteoclasts. In contrast, despite the presence of RANKL induction, the fluorescence intensity of NFATc1 was substantially decreased in SAHA-treating groups (0.5 μM and 1 μM), which was concomitant with a decline in the area and nuclear number of osteoclasts. The same treatment in BMDM also led to the same results (Fig. 4H-J). Thus, it can be concluded that SAHA treatment attenuates NFATc1 activity, which subsequently affects the expression of downstream osteoclast function-related proteins, thus retarding osteoclast differentiation and impairing bone resorbing function.

### SAHA blocks RANKL-induced activation of NF-κB and MAPK signaling cascades

**Fig. 5:**
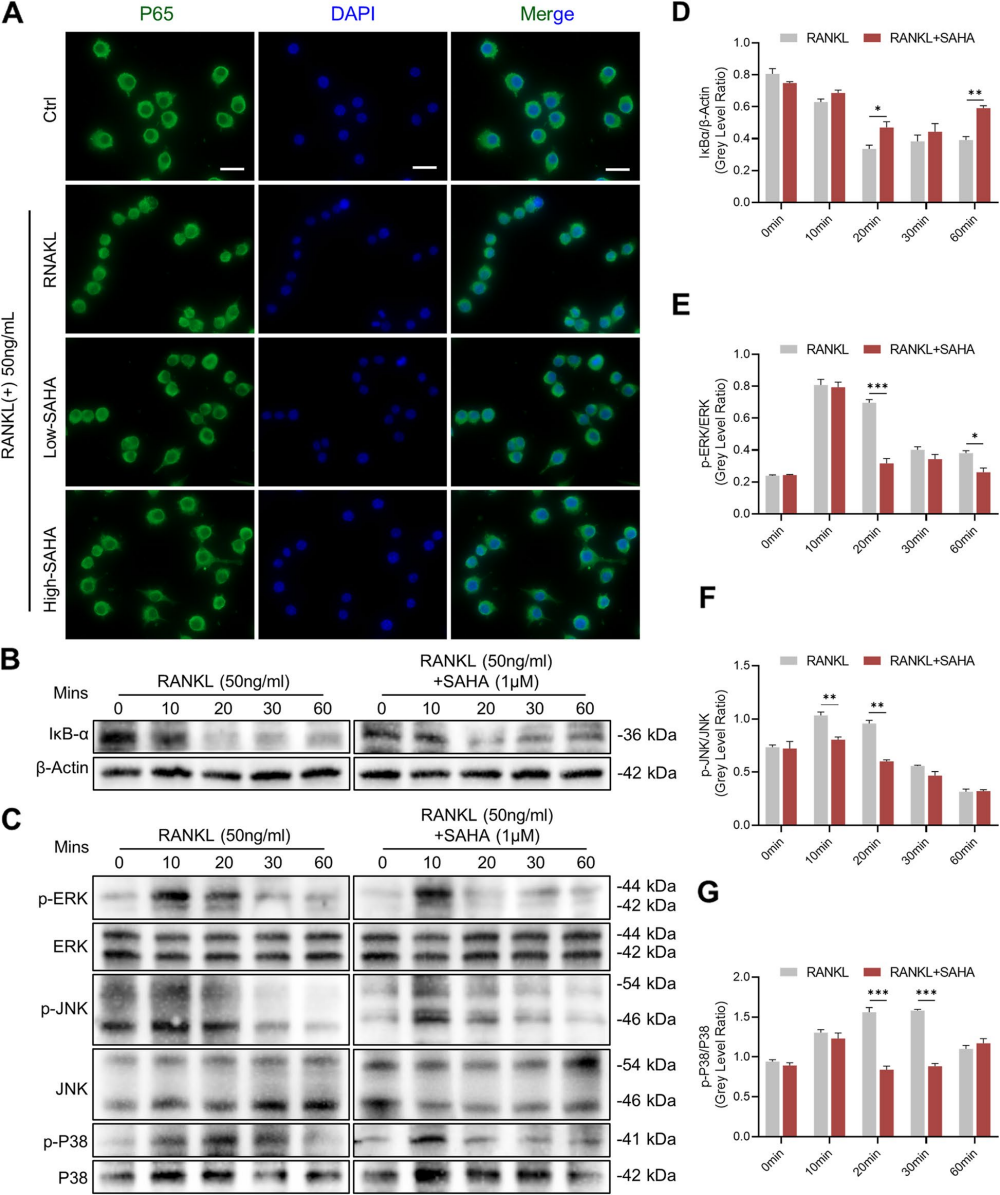
SAHA blocks RANKL-activated NF-κB and MAPK signaling pathways during osteoclastogenesis. **A** Representative fluorescence micrographs for nuclear translocation of P65 (Scale bar = 20 μm). P65 (green color) and nuclei (blue color). **B** Representative Western Blot images showing the influence of SAHA on the RANKL-mediated IκB-α degradation. **C** The phosphorylation levels of ERK, JNK and P38. **D** Quantification of IκB-α expression standardized to β-actin. **E-G** The proportion of phosphorylated ERK, JNK and P38 with respect to total ERK, JNK and P38. All data are presented as mean ± SD, *n* = 3 per group. *p < 0.05, **p < 0.01, ***p < 0.001

NFATc1 transcriptional activity relies on the activation of NF-κB and MAPK signal transduction systems. To further elucidate the underlying molecular mechanism by which SAHA suppresses RANKL-stimulated osteoclast differentiation, the activities of NF-κB and MAPK signaling cascades were examined. First, immunofluorescence staining results showed that SAHA intervention had a negative impact on nuclear translocation of P65 since it prevented RANKL-induced accumulation of P65 into nuclei (Fig. 5A). Consistent with the suppression of p65 nuclear translocation, the results of western blot showed that SAHA delayed IκB-α degradation and accelerated its restoration of expression levels, which posed a challenge to NF-κB pathway activation (Fig. 5B and D). For the MAPK pathway, we examined the protein phosphorylation of extracellular signal-regulated kinases (ERK), c-Jun N-terminal kinase (JNK) and P38 at 0, 10, 20, 30, and 60 min (Fig. 5C). After SAHA addition, the phosphorylation levels of ERK, JNK and P38 relative to their total protein levels strikingly declined within 60 mins. More precisely, as depicted in Fig. 5E–G, ERK phosphorylation was mainly impaired from 20 to 60 min, with the most pronounced suppression observed at 20 min following RANKL induction. SAHA also potently inhibited JNK and P38 phosphorylation at the early stage after 10 – 30 min of RANKL stimulation. To sum up, these data demonstrated that the adverse regulation of SAHA on osteoclastogenesis is achieved by, at least in part, interfering with the activation of NF-κB and MAPK signaling cascades.

### A potential disruption of RANK-RANKL binding mediated by SAHA

**Fig. 6:**
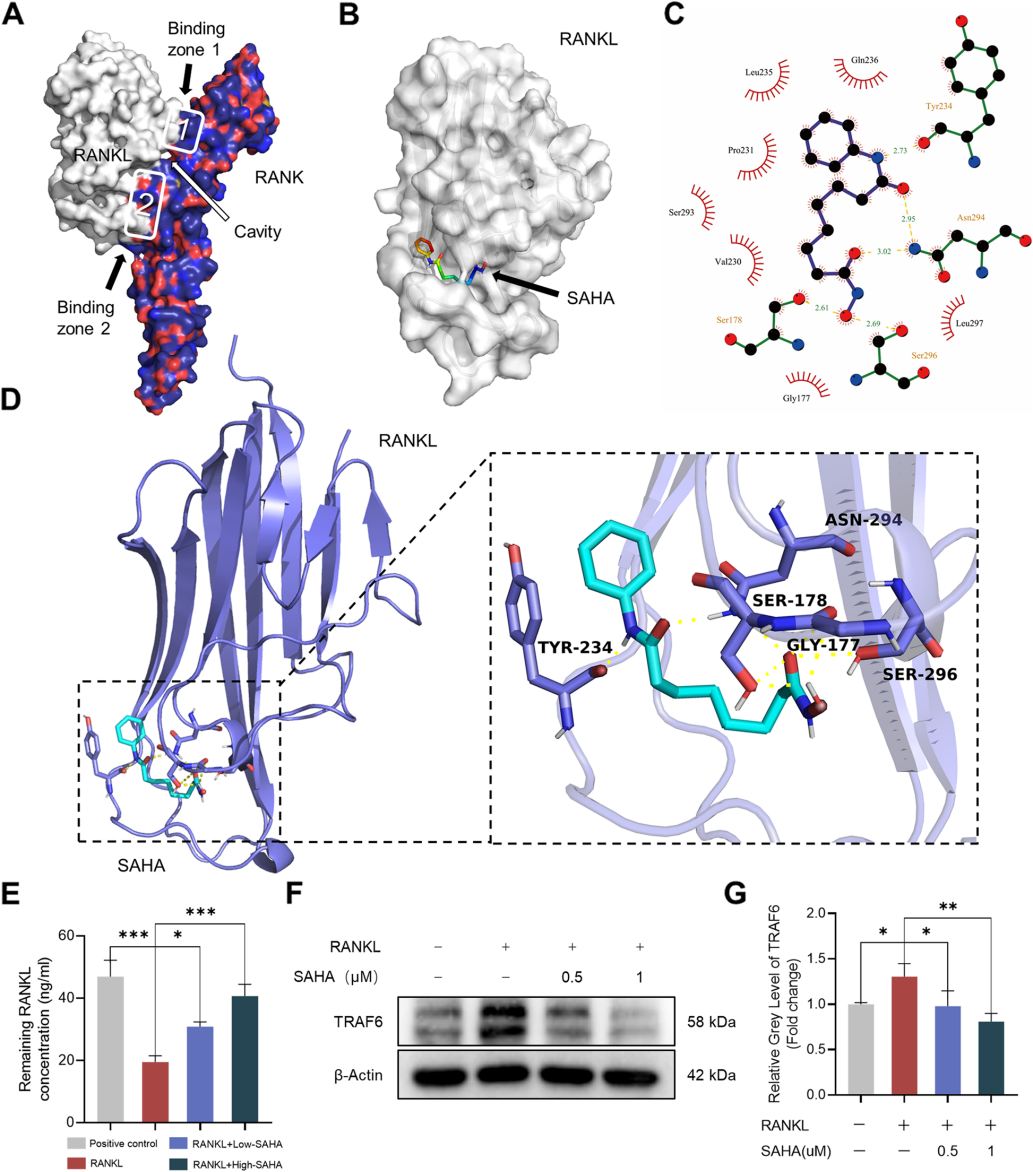
Molecular docking results of the SAHA–RANKL interaction. **A** Structural 3D image of RANK/RANKL complex. **B** Diagram of the binding capacity of molecular SAHA to the cavity on the RANKL protein surface. **C** A 2D interaction diagram of compound SAHA with RANKL. Molecular SAHA is surrounded by amino acids. The red arcs represent the sites where the protein amino acids form hydrophobic interactions with SAHA, while the sites forming hydrogen bonds exhibit complete amino acid structures. **D** Image in secondary structure indicating hydrogen bond interactions between SAHA and amino acid residues of RANKL. **E** RANKL concentration in cell culture supernatants after incubation with different doses of SAHA (0, 0.5, 1 μM) at the initial RANKL concentration of 50 ng/ml. **F** Representative images of western blotting for TRAF6 expression. **G** Quantitative analysis of TRAF6 normalized to β-actin. All data are shown as mean ± SD, *n* = 3 per group. *p < 0.05, **p < 0.01, ***p < 0.001

The protein–protein interaction between RANKL and its receptor RANK in osteoclast precursors is prerequisite for the activation of NF-κB and MAPK pathways. Given that SAHA blocked RANKL-induced canonical signaling pathways, we speculated that SAHA might directly blunt RANKL–RANK interaction and arrest several signal events. A crystal model of the RANKL–RANK complex was constructed as a basic template for SAHA matching (Fig. 6A). Computational docking suggested a high affinity between RANKL and SAHA and identified a prospective binding site for SAHA (Fig. 6B). As predicted, SAHA could bind to the active pocket (cavity on the surface) of the RANKL protein well with the lowest binding free energy of – 4.80 Kcal/mol. As shown in Fig. 6C, D, the decisive area for SAHA docking began at residue TYR234 and finally extended toward residue SER296. Next, to further verify whether SAHA induced a potential disruption of the binding of RANKL to RANK, we performed ELISA assay to quantify the extracellular RANKL added to the culture medium of RAW 264.7 cells. RANKL solutions not involved in cell incubation were used as positive controls. As a result, the abundant RANK on the cell membranes efficiently neutralized extracellular RANKL compared with positive controls, while such binding was markedly inhibited upon SAHA presence (Fig. 6E). Additionally, the interaction of RANKL and RANK contributes to initiate the recruitment of TRAF6, which subsequently evokes the activation of downstream signaling cascades. The results of western blotting indicated that SAHA intervention significantly reversed the TRAF6 overexpression in response to RANKL exposure (Fig. 6F, G). Taken together, SAHA potentially destabilized the interaction between RANKL and membrane-bound RANK and downregulates TRAF6 expression, which results in a blockade of NF-κB and MAPK signaling pathways.

### SAHA ameliorates ovariectomy-induced bone loss

**Fig. 7:**
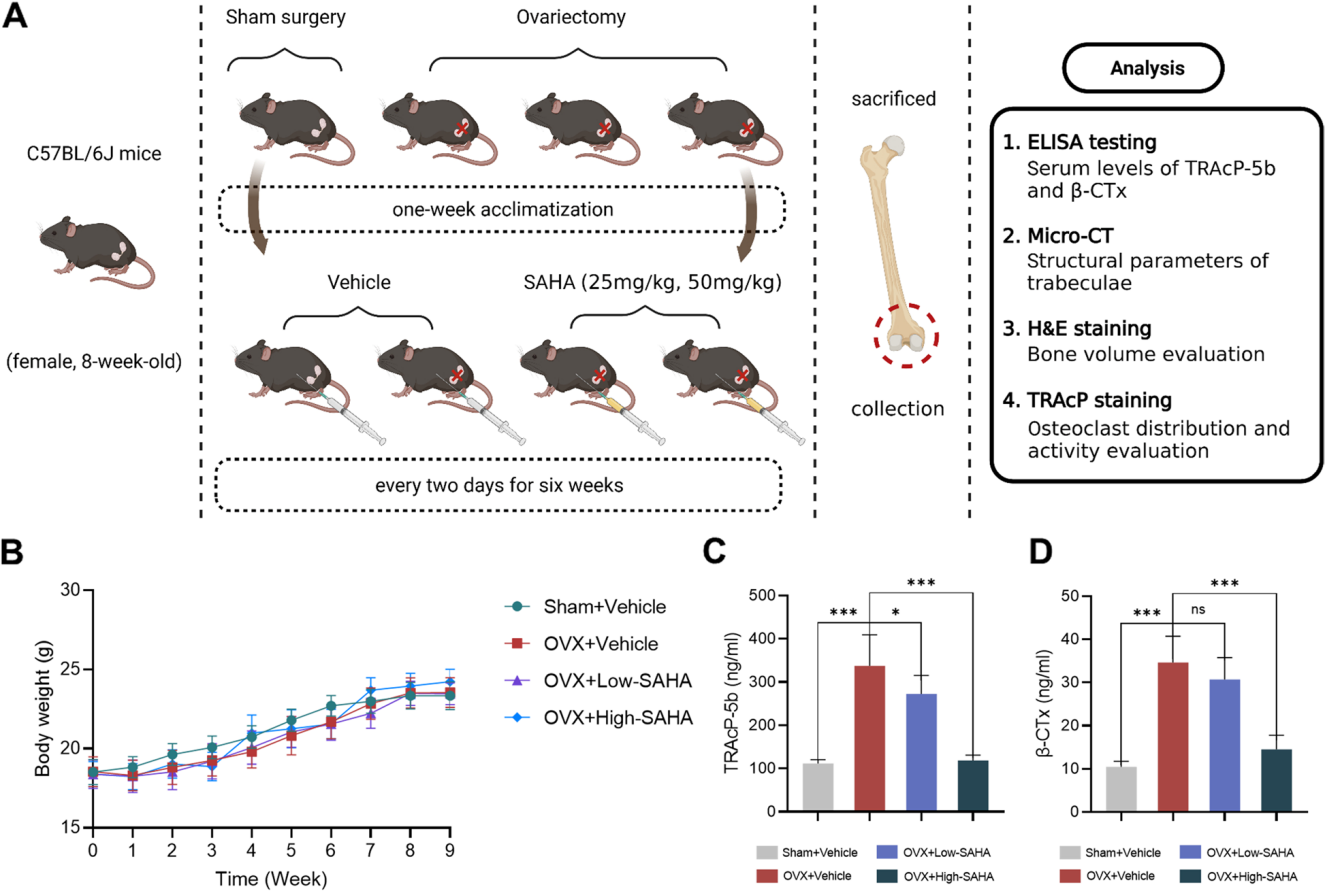
Administration of SAHA ameliorates ovariectomy-induced bone loss in mice. **A** Schematic diagram for the in vivo experiments. **B** Body weight variations of mice in each group during the period of surgery and intraperitoneal SAHA injection. **C-D** Quantitative statistics of the serum levels of bone resorption markers TRAcP-5b and β-CTx. Bar graphs are shown as mean ± SD, *n* = 6 per group. *p < 0.05, **p < 0.01, ***p < 0.001 and ns: no significance, relative to the sham-operated group and OVX-untreated group

We established an OVX model mimicking PMOP by surgically removing the ovaries of mice to assess the therapeutic efficacy of SAHA on bone loss in vivo. Different dilutions of SAHA (25 mg/kg and 50 mg/kg) or vehicle were intraperitoneally administered into OVX mice every two days for six weeks prior to sacrifice euthanasia (Fig. 7A). No unexpected severe adverse events or mortality were recorded during the trial. Moreover, the results demonstrated that SAHA had no observable side effects on bodyweight (Fig. 7B) or significant toxicity on vital organs (heart and lung) and major metabolic organs (liver and kidney) in treated mice versus non-treated mice (Additional file 3: Fig. S2).

**Fig. 8:**
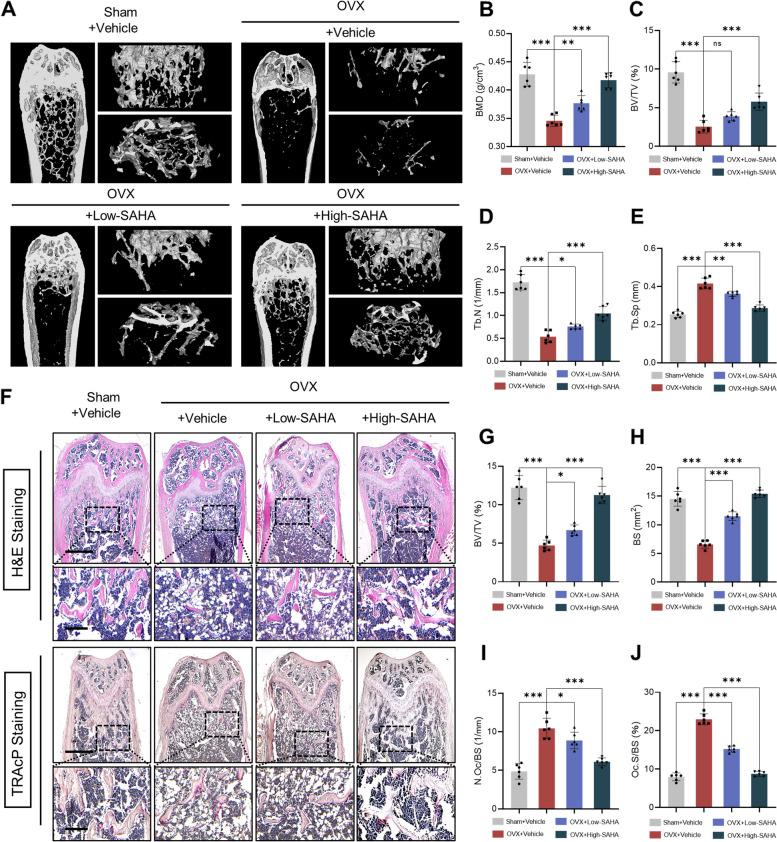
Administration of SAHA ameliorates ovariectomy-induced bone loss in mice. **A** Representative 3D images of distal femurs in each group were reconstructed by Micro-CT scanner. **B-E** Quantitative statistics of trabecular morphological parameters, including BMD, BV/TV, Tb. N and Tb.Sp. **F** Representative images of distal femurs sections in each group stained with H&E and TRAcP (Scale bar = 400 μm; scale bar = 100 μm in the enlarged images). **G-J** Quantitative statistics of BV/TV, BS, N.Oc/BS, and Oc.S/BS. All data are shown as mean ± SD, *n* = 6 per group. *p < 0.05, **p < 0.01, ***p < 0.001 and ns: no significance, relative to the sham-operated group and OVX-untreated group

SAHA administration, especially at a high dose of 50 mg/kg, reduced the serum levels of the bone resorption markers TRAcP-5b and β-CTx in OVX mice (Fig. 7 C, D). Micro-CT scan reconstruction demonstrated that treatment with SAHA alleviated estrogen deficiency-induced bone loss and trabecular structural destruction (Fig. 8A). Through quantification of trabecular morphological parameters, we found a decrease in BMD, BV/TV and Tb. N as well as an increase in Tb. Sp in the OVX-untreated group, while these changes were significantly reversed after SAHA treatment, especially in the OVX + High-SAHA group (Fig. 8B-E). Representative 3D image reconstruction of cortical bone in OVX mice was depicted in Additional file 4: Fig. S3A. The quantitative analysis revealed no statistically significant differences in cortical bone parameters comprising BMD, Ct. Th and Ct. Ar between the OVX-untreated group and SAHA-treated group (Additional file 4: Fig. S3B–D). H&E staining labeled the intramedullary trabecular bone structure for histomorphometric evaluation, whose data validated the above micro-CT scanning results (Fig. 8F–H). Additionally, in line with TRAcP staining in vitro, the results of TRAcP staining performed on bone slices of isolated femurs confirmed that SAHA administration dramatically reduced the osteoclast surface (Oc. S/BS) and osteoclast counts (N.Oc/BS) relative to the OVX group (Fig. 8I, J). Collectively, these findings indicate that SAHA has anti-osteoporosis properties in vivo, which rescues estrogen deficiency-induced bone loss by restricting the formation and activity of osteoclasts.

To further investigate the mechanism by which SAHA ameliorated ovariectomized-induced bone loss, we performed IHC to determine the expression of TRAF6, p-P65, and p-ERK in the femoral tissues of mice. As shown in Additional file 5: Fig. S4, positive expression of TRAF6, p-P65, and p-ERK in the distal femur was significantly increased in OVX group compared with sham group, whereas these changes were rescued by SAHA in a dose-dependent manner. Therefore, consistent with the results of in vitro experiments, SAHA interfered with TRAF6 recruitment and regulates the activation of NF-κB and MAPK signaling pathways in vivo*,* which consequently results in a reduction in the formation of multinucleated bone-resorbing osteoclasts.

## Discussion

Osteoporosis is the most widespread orthopedic disorder in the elderly population, whose onset is mainly driven by accelerated bone loss as a result of excessive activation of osteoclasts [8]. Thus far, despite the presence of several anti-osteoporosis therapies targeting osteoclast-mediated bone resorption, including bisphosphonates, teriparatide, denosumab, etc., the demand for long-term use and severe adverse effects like atypical femoral fractures and jaw osteonecrosis have limited the wide clinical applications of these drugs [11, 12]. Hence, the search for new corresponding substitutes that repress osteoclast function is always warranted for the amelioration of osteoporosis therapy. In view of the broad-spectrum therapeutic activity manifested by SAHA, we sought to shed light on whether SAHA could serve as a potential candidate for the management of osteoporosis.

In the present work, we first demonstrated that SAHA suppressed RANKL-stimulated osteoclast differentiation in a dose-dependent manner in vitro. Analogously, a previous study reported that SAHA could inhibit human osteoclastic bone resorption in vitro at low nanomolar concentrations [28]. However, the potential molecular mechanisms by which SAHA abolished osteoclastogenesis need to be further explored. TRAcP staining data indicated a pronounced inhibitory effect by SAHA treatment mainly in the early phase of osteoclast differentiation. Intriguingly, our results of RNA-seq further revealed an altered transcriptome profile of SAHA-treated osteoclasts, with a range of genes relevant to osteoclasts identified to be downregulated. These genes comprised *Nfatc1*, *Atp6v0d2*, and *Acp5* in precursor osteoclasts, *Src* in mature osteoclasts, as well as *Ctsk*, *Mmp9*, *Slc4a2* and *Tcirg1* in resorbing osteoclasts, suggesting that SAHA might govern osteoclast formation and function throughout all periods of osteoclast development.

Our findings suggest that SAHA negatively regulated the activity of NF-κB and MAPK signaling cascades and subsequently retarded the expression levels of specific osteoclast-related markers. The interaction of RANKL and RANK represents a key initiating event for the activation of a series of downstream targets, including NF-κB and MAPK pathways [14]. We revealed the strong binding affinity between SAHA and RANKL which was based on their complex non-covalent interactions, as indicated by molecular docking. ELISA analysis showed a remarkable inhibitory effect of SAHA on the binding of extracellular RANKL to membrane-bound RANK. The interaction between RANK and RANKL recruits TRAF6 to initiate downstream signaling cascades, and the reduction of TRAF6 protein levels after SAHA treatment further reinforce our hypothesis. Therefore, we proposed that SAHA retarded the activity of classical RANKL-induced signaling via blunting RANKL-RANK interaction.

Although we preliminarily confirmed the potential disruption of RANK-RANKL binding and abrogation of TRAF6 recruitment after treating SAHA, further targeted protein binding experiments are still warranted to validate the specific drug-protein binding sites. For instance, biolayer interferometry (BLI) assay or surface plasmon resonance (SPR) analysis can be employed to further support the direct interaction of SAHA with RANKL through estimating the kinetics of the phases of association and dissociation as well as the affinity [39, 40]. The protein–protein interactions between RANKL and RANK in the presence of SAHA can also been analyzed through competition analysis [41]. Furthermore, small molecular drugs appear to be rarely selective enough to interact solely with their target proteins. It is necessary to explore the structure-activity relationship (SAR) study of SAHA in osteoclast differentiation. The synthesis and screening of several derivatives might facilitate further improvement of SAHA potency and selectivity [41, 42]. RANKL exists soluble and membrane-bound forms [43]. Utilizing ELISA analysis to examine exogenous RANKL concentration in the cell culture media, we demonstrated SAHA-induced disruption of membrane RANK and extracellular RANKL binding. Huang et al. identified a selective sRANKL inhibitor that can alleviate bone loss in OVX mice and rats while avoiding membrane RANKL (mRANKL) mediated immunosuppression [44]. Controversially, despite the upregulation of sRANKL in the context of estrogen deficiency, the lack of sRANKL seems to fail to improve ovariectomy-induced bone loss [45]. We hope to further investigate the selectivity of SAHA for sRANKL and mRANKL in the future and validate it in more chronic bone loss experimental models. Given that sRANKL was reported to accelerate tumor metastasis to bone [46], it would be attractive to explore the effect of the anti-tumor agent SAHA in a related direction.

Of note, the disrupted RANK-RANKL interaction appears not to be the only reason for the impaired osteoclastogenesis induced by SAHA. HDACs act as important epigenetic regulators of intracellular gene expression and are emerging therapeutic targets for the treatment of diverse disorders. SAHA, the first generation HDACi FDA approved for CTCL, has led the way in the popularity of HDACi in the field of oncology therapy [47]. As mentioned above, in addition to its extensive anti-tumor activities, SAHA has shown therapeutic efficacy in a wide range of immunoinflammatory diseases such as EAU, RA, and OA [25, 48, 49]. Furthermore, the vital role of HDACs in physiological and pathological bone remodeling has been well summarized [27, 50]. Acetylation of NF-κB is a dynamic process that can be negatively regulated through HDACs [51], whereas selected HDACi have been reported to be capable of inhibiting the nuclear translocation of NF-κB p65 by altering its acetylation levels [52]. HDACi trichostatin A (TSA) suppressed RANKL-stimulated osteoclast differentiation via restraining the induction of osteoclastogenic transcription factor c-Fos [53]. TSA and sodium butyrate (NaB) were demonstrated to inactivate TNF-α-induced nuclear translocation of NF-κB and sRANKL-induced MAPK signals [52]. Another study has shown that HDACi FR901228 markedly induced the production of osteoclast inhibitory factor, IFN-β, to control osteoclast formation [54]. As a kind of pan-HDACi targeting multiple HDACs in both classes I and II [26], SAHA is supposed to participate in the modulation of bone homeostasis. As expected, this study demonstrated that SAHA negatively regulated RANKL-induced osteoclast differentiation in vitro and successfully prevented ovariectomy-induced bone loss in vivo. Nevertheless, given the intricacies of physiological activities manifested by distinct HDAC enzymes, and the precise role of individual HDACs during osteoclast differentiation that remains to be adequately elucidated, the broad-spectrum anti-HDAC activities of SAHA can lead to some potential side effects. The exact HDAC inhibition profile of SAHA might also vary with the milieu of osteoclasts under different physiological and pathological environments.

Previous study has revealed that SAHA elicited metabolic reprogramming in the context of glioblastoma (GBM) [55]. Osteoclasts tend to undergo active metabolic reprogramming to accommodate energy demands during the process of RANKL-induced osteogenesis and bone resorption [56]. Thus, SAHA has the potential to affect intrinsic osteoclast metabolic pathways like oxidative phosphorylation.

SAHA has been reported to block NF-κB signal transduction and downregulate interleukin (IL)- 1β induced IL-6 production, thereby restoring the chondrogenic potential of synovium-derived mesenchymal stem cells (SMSCs) to repair temporomandibular joint (TMJ) damage [57]. The anti-inflammatory efficiency of SAHA treatment on experimental autoimmune uveitis (EAU) is achieved in part through the suppression of the NF-κB p65 transcription factor [49]. Takada et al. has shown that SAHA treatment attenuated NF-κB signal transduction induced by multiple stimuli, such as TNF-α, IL-1β, okadaic acid, doxorubicin, and lipopolysaccharide, in different cell types [58]. It follows that the mechanism of NF-κB activation upon SAHA treatment is complicated. In this work, we found that SAHA impeded nuclear accumulation of p65, degradation of IκBα, and phosphorylation of ERK, JNK, and p38 in the presence of RANKL, which consequently repressed downstream NFATc1 signaling. We also showed a high affinity between SAHA and RANKL as well as a notable disruption of RANKL-RANK interaction. Therefore, SAHA performs essential regulatory functions during the process of osteoclast differentiation through modulating RANKL-evoked signaling. Intriguingly, the activation of NF-κB and MAPK pathways induced by various RANKL-independent mechanisms are fundamental for inflammation and immune response. Given that the chronic inflammatory microenvironment in the context of aging or estrogen deficiency exacerbates osteoclastic bone resorption [59], we speculate that the immunosuppressive property of SAHA will help optimize the management of osteoporotic bone loss.

Ovariectomy is frequently used as a standardized procedure for bone loss due to estrogen withdrawal. It mimics the characteristics of bone phenotype alterations in the process of age-related osteoporosis caused by the physiological decrease of estrogen in human females following menopause. The in vivo experimental results showed that SAHA administration effectively reduced the serum levels of bone resorption markers, ameliorated the destruction of bone trabecular microarchitecture, and suppressed hyperactive osteoclasts in ovariectomized mice. These findings provide the first evidence of the therapeutic potential of SAHA for estrogen deficiency-induced osteoporotic bone loss in vivo. Previous studies have revealed that SAHA promotes osteogenic differentiation and matrix mineralization in both MC3T3-E1 cells and mesenchymal stem cells in vitro [36, 60]. Hence, the attenuation of bone loss in OVX mice after treatment with SAHA might be mediated through bidirectional modification of osteoclastic bone resorption and osteoblastic bone formation. Moreover, the anti-osteoporotic activity of SAHA in an ovariectomized mouse model was observed at appropriate dose via intraperitoneal injection. Despite histological analysis confirming drug safety in vivo, the current use of SAHA still represents extremely high doses. Continued optimization of SAHA administration regimens is necessary to avoid unwanted off-target effects in the long-term treatment of patients with osteoporosis.

## Conclusions

**Fig. 9:**
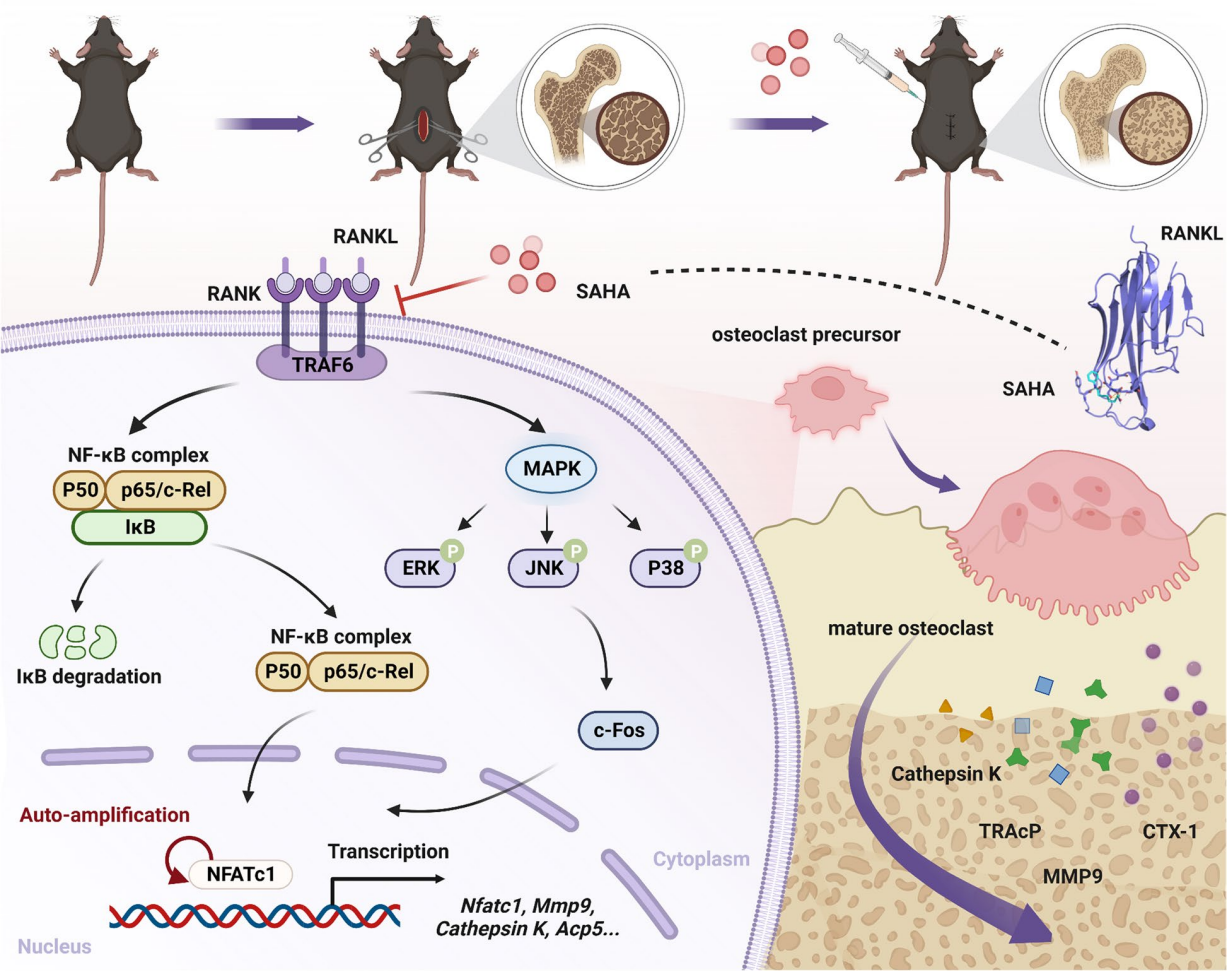
Proposed schematic diagram for SAHA suppression on RANKL-induced osteoclastogenesis. The high affinity between SAHA and RANKL results in blockade of RANKL-RANK interaction, thereby interfering with RANKL-induced signaling cascades and osteoclastic bone resorption. Additionally, SAHA administration effectively ameliorates ovariectomy-induced bone loss in mice

To summarize, SAHA dose-dependently retarded RANKL-induced osteoclastogenesis in vitro and efficiently attenuated ovariectomy-induced bone loss in vivo. Mechanistically, SAHA interfered with the NF-κB and MAPK signaling pathways and downstream NFATc1 activity at least partly through a targeted blockade of RANKL-RANK interaction (Fig. 9). Based on the above findings, we explored a novel drug application for the classical pan-HDACi SAHA, which may serve as a prospective alternative therapeutic option for osteoporosis or osteoclast-related bone disorders.

### Supplementary Information

**Supplementary material 1.**

**Supplementary material 2.**

**Supplementary material 3.**

**Supplementary material 4.**

**Supplementary material 5.**

**Supplementary material 6.**

## Data Availability

No datasets were generated or analysed during the current study.

## Abbreviations

PMOP: Postmenopausal osteoporosis
SAHA: Vorinostat, suberoylanilide hydroxamic acid
TRAcP: Tartrate-resistant acid phosphatase
NF-κB: Nuclear factor κB
MAPK: Mitogen-activated protein kinases
RANKL: Receptor activator of nuclear factor κB ligand
TRAF6: TNF receptor associated factor 6.
NFATc1: Nuclear factor of activated T cells c1
HDACi: Histone deacetylase inhibitor
DMSO: Dimethyl sulfoxide
FBS: Fetal bovine serum
CCK-8: Cell Counting Kit-8
PFA: Paraformaldehyde
SEM: Scanning electron microscope
KEGG: Kyoto Encyclopedia of Genes and Genomes
GO: Gene Ontology
GSEA: Gene set enrichment analysis
qRT-PCR: Quantitative real-time polymerase chain reaction
ELISA: Enzyme-linked immunosorbent assay
BMD: Bone mineral density
BV/TV: the trabecular bone volume to total bone volume ratio
Tb.N: Trabecular number
Tb.Sp: Trabecular separation
Ct.Th: Cortical thickness
Ct.Ar: Cortical bone area
ERK: Extracellular signal-regulated kinases
JNK: c-Jun N-terminal kinase
β-CTx: type I collagen cross-linked C-terminal telopeptide
