# Supplementary material for: Pan-histone deacetylase inhibitor vorinostat suppresses osteoclastic bone resorption through modulation of RANKL-evoked signaling and ameliorates ovariectomy-induced bone loss
Source: Cell Commun Signal. 2024 Mar 4;22:160. doi: 10.1186/s12964-024-01525-w (PMC10913587; doi:10.1186/s12964-024-01525-w)
Supplement: Supplementary file 1 — Supplementary material 1. [file 12964_2024_1525_MOESM1_ESM.docx]

**Table S1. Primer sequences for osteoclast-specific genes**

| Gene (mus) | Forward Primers (5’-3’) | Reverse Primers (5’-3’) |
| --- | --- | --- |
| *Nfatc1* | GAGAATCGAGATCACCTCCTAC | TTGCAGCTAGGAAGTACGTCTT |
| *Mmp9* | CAAAGACCTGAAAACCTCCAAC | CAAAGACCTGAAAACCTCCAAC |
| *Ctsk* | GCTTGGCATCTTTCCAGTTTTA | CAACACTGCATGGTTCACATTA |
| *c-Fos* | TCTCTAGTGCCAACTTTATCCC | GAGATAGCTGCTCTACTTTGCC |
| *Acp5* | CAAGAACTTGCGACCATTGTTA | ATCCATAGTGAAACCGCAAGTA |
| *Oscar* | ACTGTTGCTATTACCACACGCCTTC | CCATTCCTGCCAGCCCCAAAC |
| *Actb* | CTACCTCATGAAGATCCTGACC | CACAGCTTCTCTTTGATGTCAC |
