# Supplementary material for: Pan-histone deacetylase inhibitor vorinostat suppresses osteoclastic bone resorption through modulation of RANKL-evoked signaling and ameliorates ovariectomy-induced bone loss
Source: Cell Commun Signal. 2024 Mar 4;22:160. doi: 10.1186/s12964-024-01525-w (PMC10913587; doi:10.1186/s12964-024-01525-w)
Supplement: Supplementary file 2 — Supplementary material 2. [file 12964_2024_1525_MOESM2_ESM.docx]

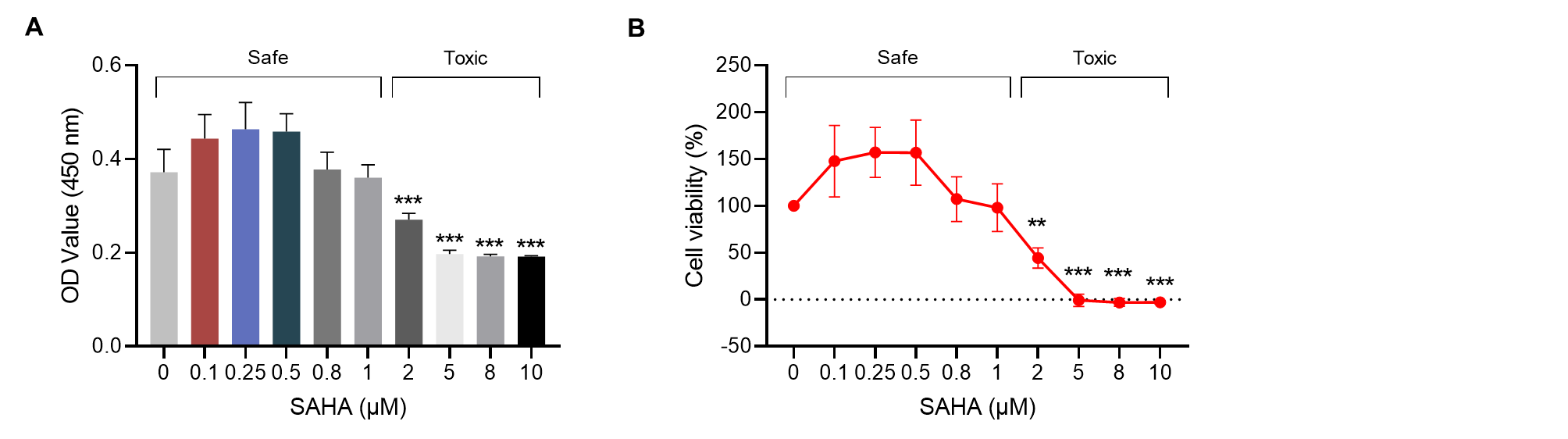


**Fig. S1 Inhibitory and cytotoxicity effects of SAHA in BMDM. A-B** The effects of distinct SAHA doses on BMDM cell viability for 72 h as evaluated by CCK-8 assay. Bar graphs are shown as mean ± SD. *p < 0.05, **p < 0.01, ***p < 0.001, as compared to the control group treated with 0 μM SAHA.
