# Supplementary material for: Pan-histone deacetylase inhibitor vorinostat suppresses osteoclastic bone resorption through modulation of RANKL-evoked signaling and ameliorates ovariectomy-induced bone loss
Source: Cell Commun Signal. 2024 Mar 4;22:160. doi: 10.1186/s12964-024-01525-w (PMC10913587; doi:10.1186/s12964-024-01525-w)
Supplement: Supplementary file 3 — Supplementary material 3. [file 12964_2024_1525_MOESM3_ESM.docx]

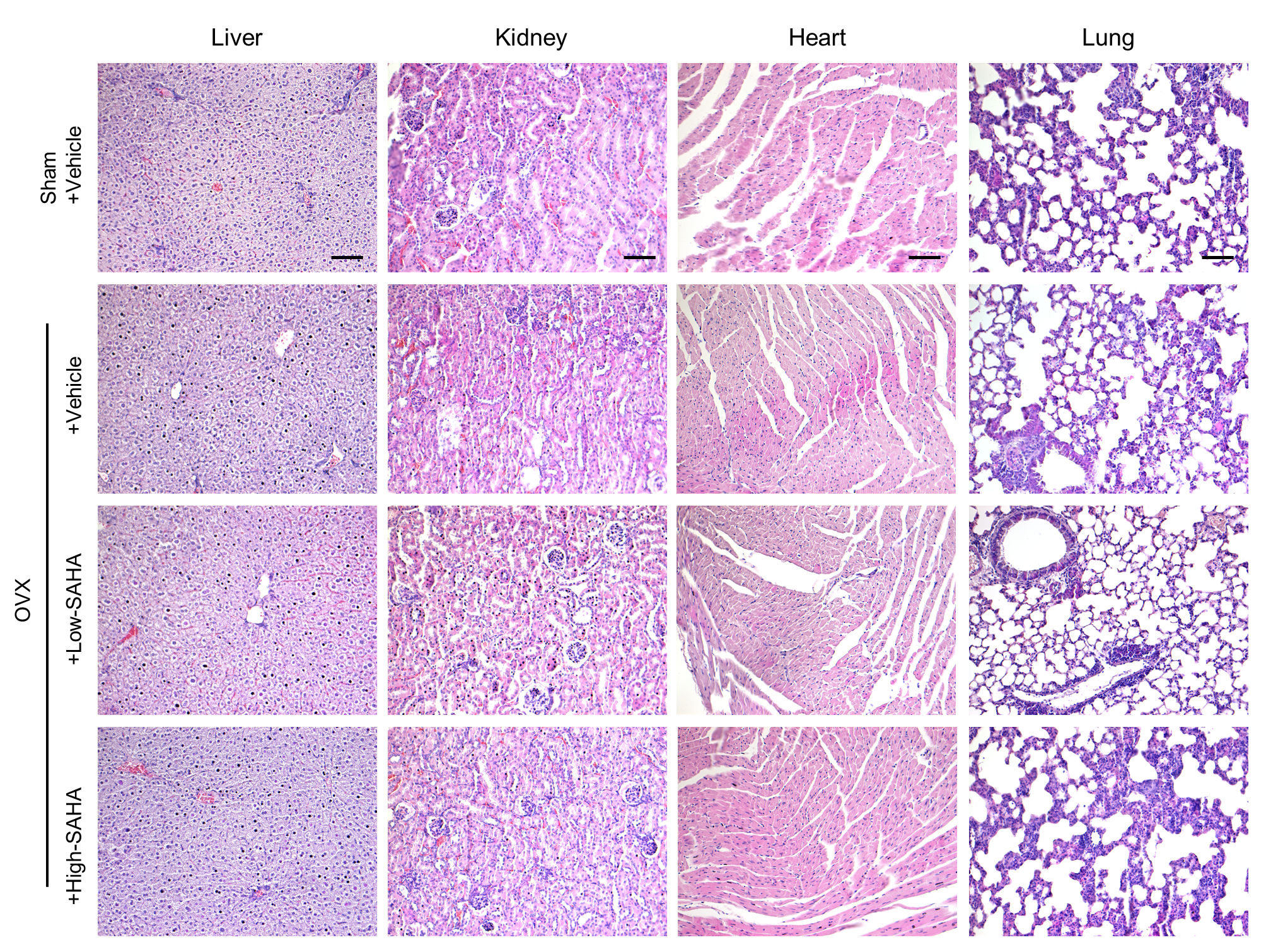


**Fig. S2 Toxic effect of SAHA on visceral organs.** Representative H&E staining of the Liver, kidney, heart and lung tissues in each group (Scale bar = 100 μm).
