# Supplementary material for: Pan-histone deacetylase inhibitor vorinostat suppresses osteoclastic bone resorption through modulation of RANKL-evoked signaling and ameliorates ovariectomy-induced bone loss
Source: Cell Commun Signal. 2024 Mar 4;22:160. doi: 10.1186/s12964-024-01525-w (PMC10913587; doi:10.1186/s12964-024-01525-w)
Supplement: Supplementary file 4 — Supplementary material 4. [file 12964_2024_1525_MOESM4_ESM.docx]

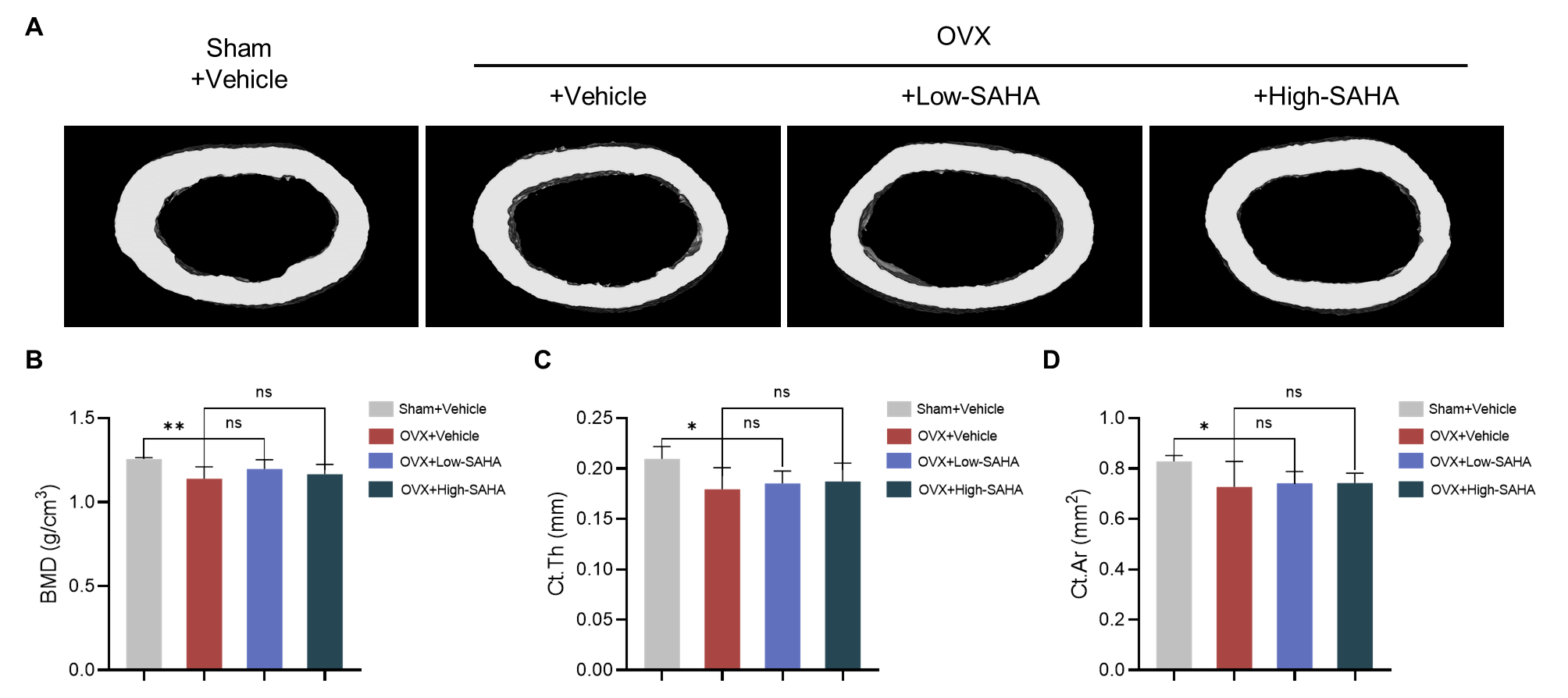


**Fig. S3 Effect of SAHA on cortical bone of the distal femur in OVX mice. A** Representative Micro-CT images of distal femur cortical bone in each group. **B-D** Quantification of cortical bone-associated microstructural parameters (BMD, Ct.Th and Ct.Ar). Bar graphs are presented as mean ± SD, n = 6 per group. *p < 0.05, **p < 0.01 and ns: no significance, relative to the sham-operated group and OVX-untreated group. BMD, bone mineral density; Ct.Th, cortical thickness; Ct.Ar, cortical bone area; OVX, ovariectomized.
