# Supplementary material for: Pan-histone deacetylase inhibitor vorinostat suppresses osteoclastic bone resorption through modulation of RANKL-evoked signaling and ameliorates ovariectomy-induced bone loss
Source: Cell Commun Signal. 2024 Mar 4;22:160. doi: 10.1186/s12964-024-01525-w (PMC10913587; doi:10.1186/s12964-024-01525-w)
Supplement: Supplementary file 5 — Supplementary material 5. [file 12964_2024_1525_MOESM5_ESM.docx]

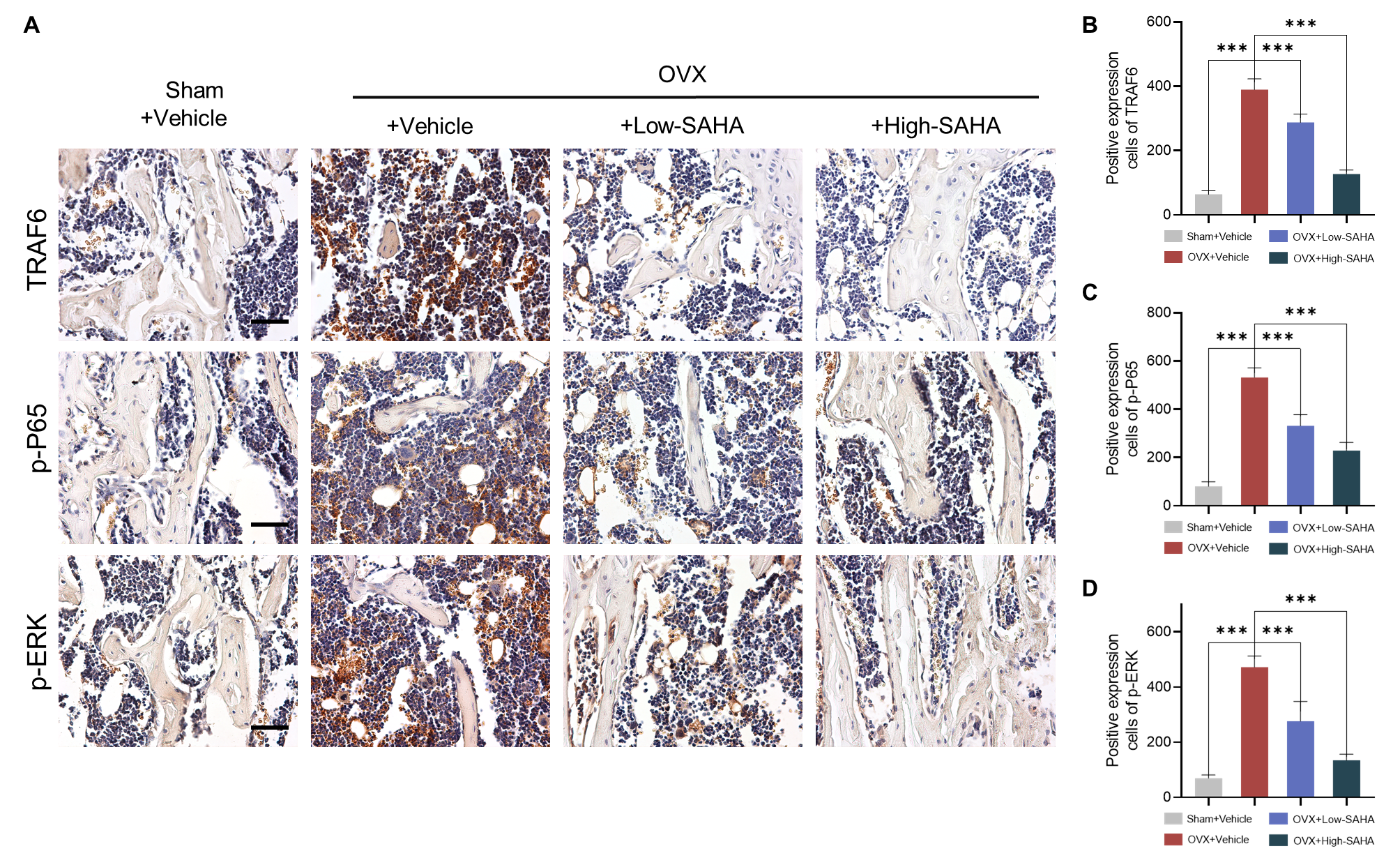


**Fig. S4 Effect of SAHA on RANKL-induced signaling in the distal femur of OVX mice. A** Representative photomicrograph of TRAF6, p-P65, and p-ERK expression in the femoral tissues of OVX mice stained by immunohistochemistry. (Scale bar = 50 μm). **B-D** Statistical analysis of positive expression cells of TRAF6, p-P65 and p-ERK. Bar graphs are presented as mean ± SD, n = 6 per group. ***p < 0.001, relative to OVX + Vehicle group.
