# Supplementary material for: Pan-histone deacetylase inhibitor vorinostat suppresses osteoclastic bone resorption through modulation of RANKL-evoked signaling and ameliorates ovariectomy-induced bone loss
Source: Cell Commun Signal. 2024 Mar 4;22:160. doi: 10.1186/s12964-024-01525-w (PMC10913587; doi:10.1186/s12964-024-01525-w)
Supplement: Supplementary file 6 — Supplementary material 6. [file 12964_2024_1525_MOESM6_ESM.docx]

**The whole un-cropped images of the original western blots from figure 4.**

**
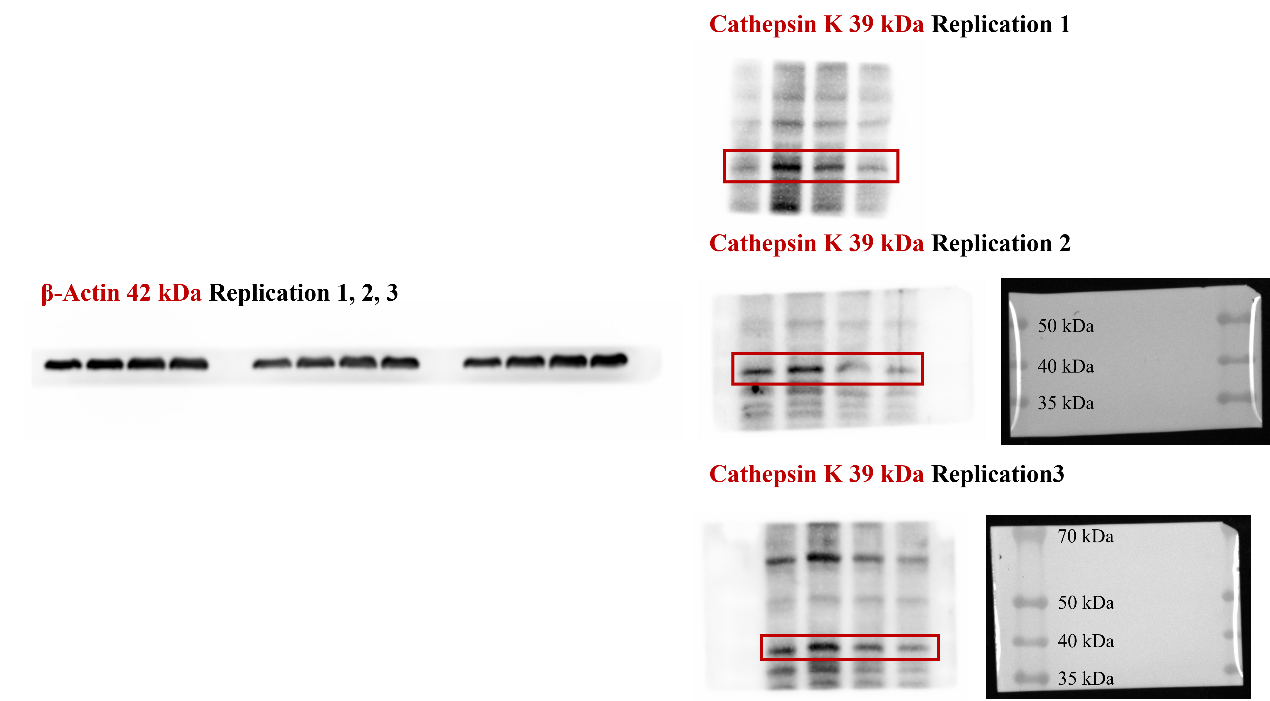
**

**
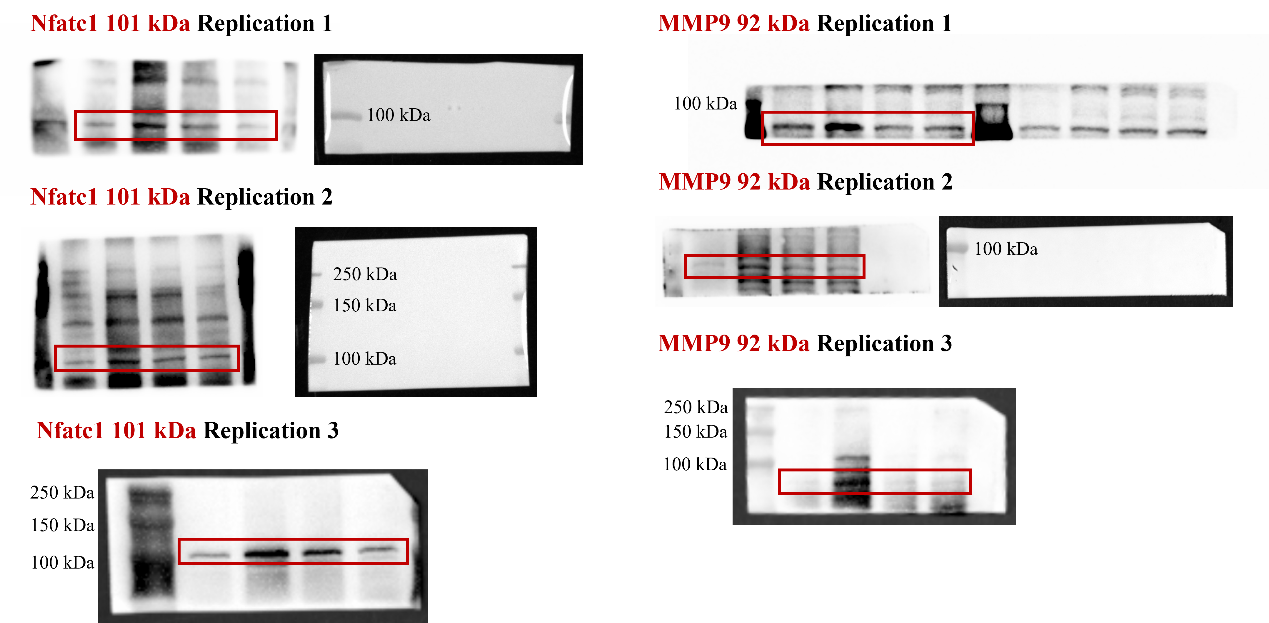
**

**The whole un-cropped images of the original western blots from figure 5.**

**
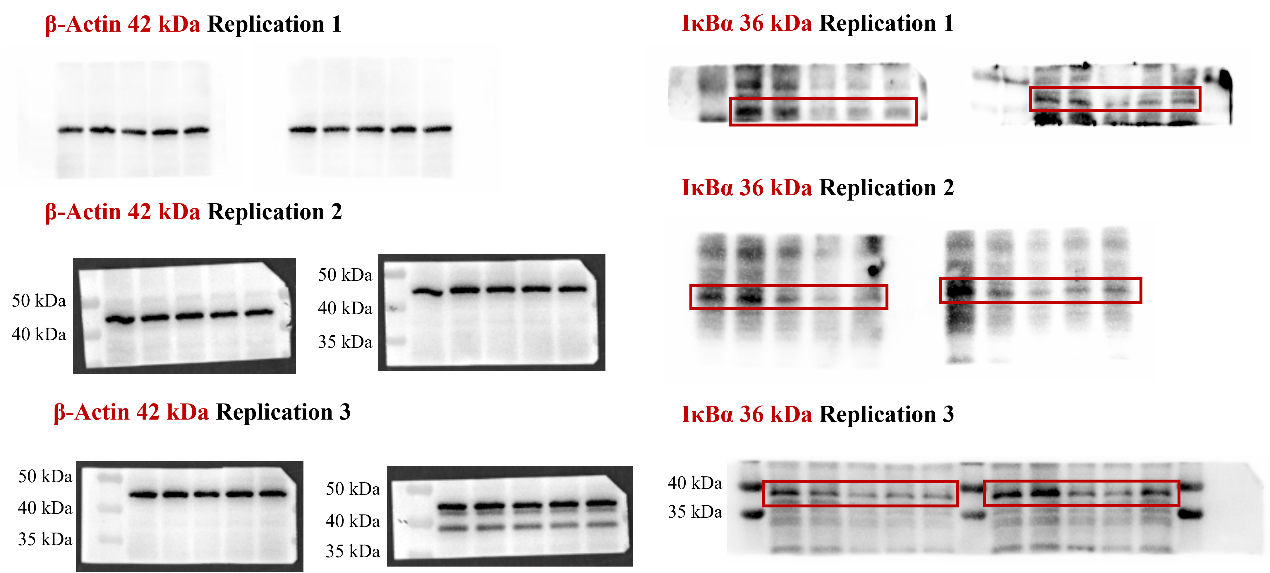
**

**
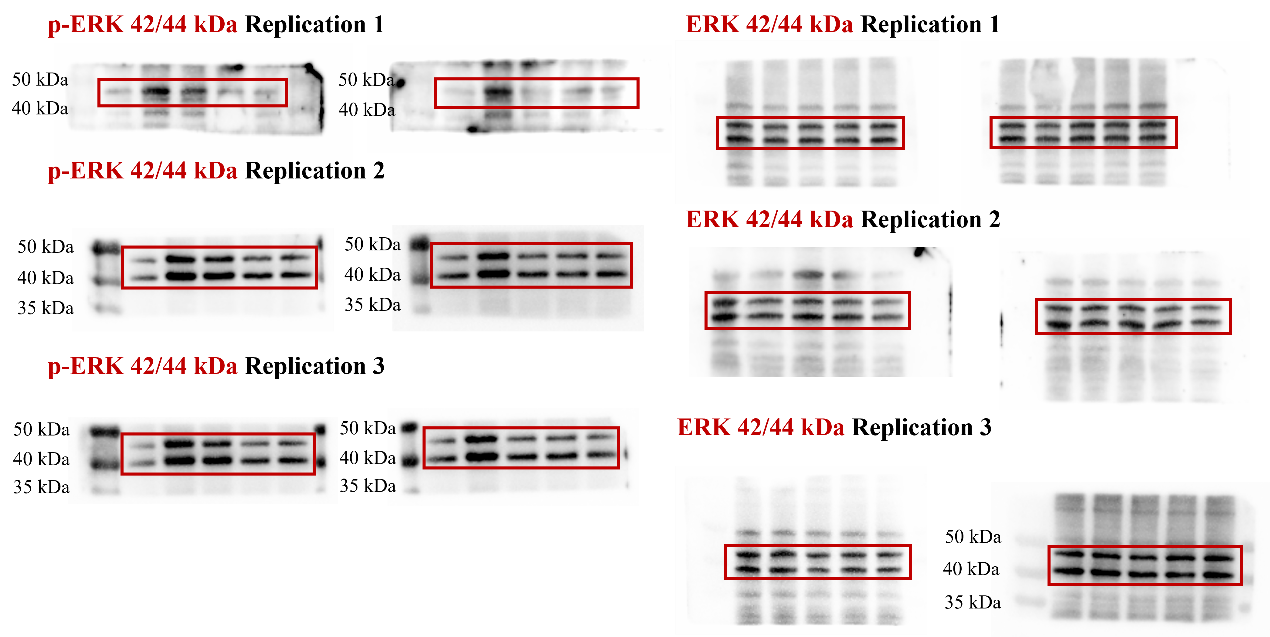
**

**
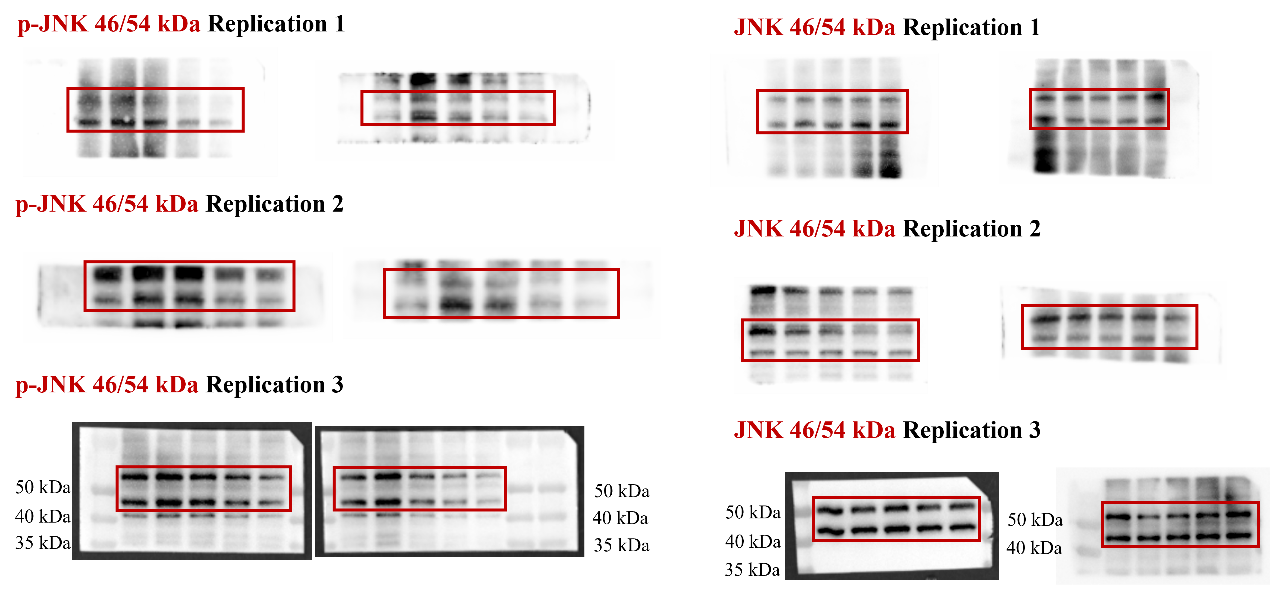
**

**
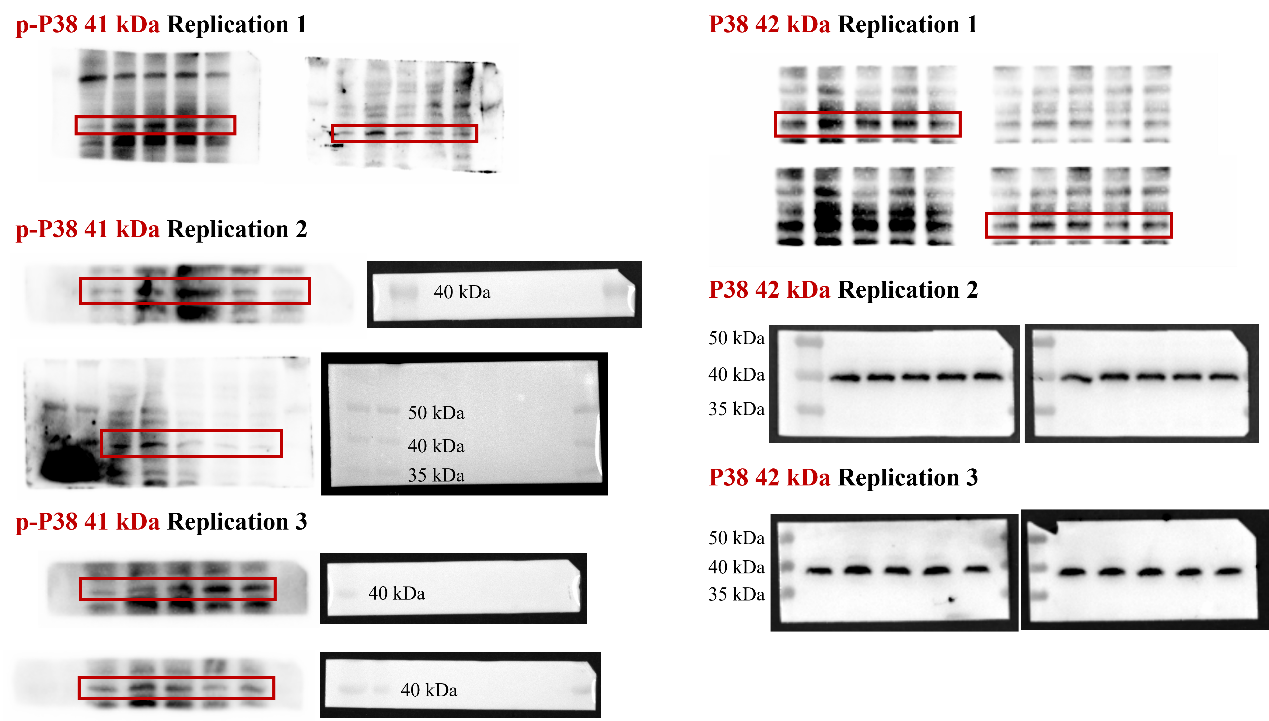
**

**The whole un-cropped images of the original western blots from figure 6.**

**
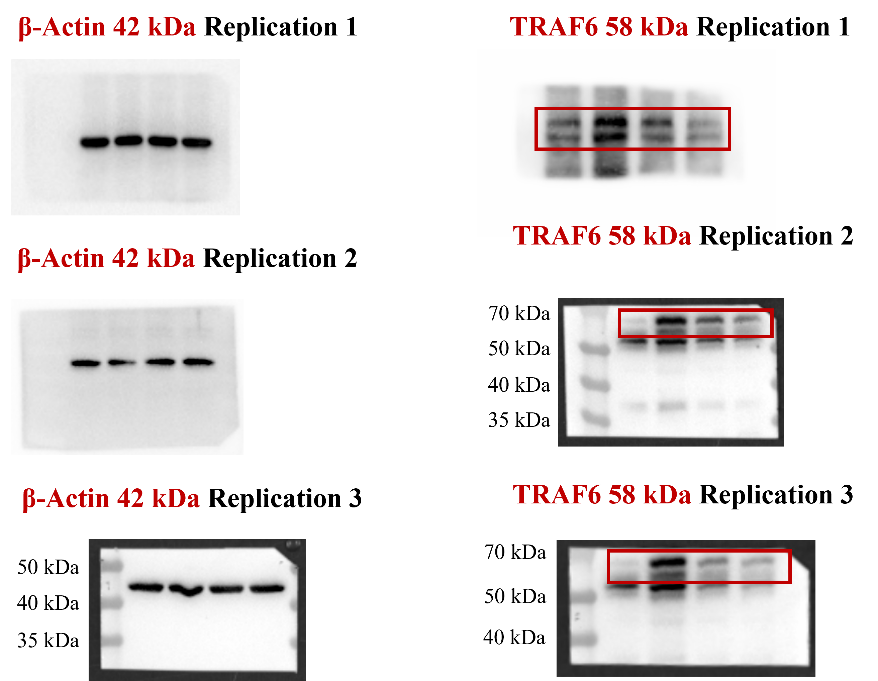
**
